# In vivo phosphoproteomics reveals kinase activity profiles that predict treatment outcome in triple-negative breast cancer

**DOI:** 10.1038/s41467-018-05742-z

**Published:** 2018-08-29

**Authors:** Ivana Zagorac, Sara Fernandez-Gaitero, Renske Penning, Harm Post, Maria J. Bueno, Silvana Mouron, Luis Manso, Manuel M. Morente, Soledad Alonso, Violeta Serra, Javier Muñoz, Gonzalo Gómez-López, Jose Francisco Lopez-Acosta, Veronica Jimenez-Renard, Albert Gris-Oliver, Fatima Al-Shahrour, Elena Piñeiro-Yañez, Jose Luis Montoya-Suarez, Juan V. Apala, Amalia Moreno-Torres, Ramon Colomer, Ana Dopazo, Albert J. R. Heck, Maarten Altelaar, Miguel Quintela-Fandino

**Affiliations:** 1 Breast Cancer Clinical Research Unit, CNIO – Spanish National Cancer Research Center, 28029, Madrid, Spain; 2 Biomolecular Mass Spectrometry and Proteomics, Bijvoet Center for Biomolecular Research and Utrecht Institute for Pharmaceutical Sciences, Utrecht University, Padualaan 8, 3584 CH Utrecht, The Netherlands; 3 Netherlands Proteomics Center, Padualaan 8, 3584 CH Utrecht, The Netherlands; 4 Medical Oncology, Hospital 12 de Octubre, Madrid, 28029 Spain; 5 Biobank, CNIO – Spanish National Cancer Research Center, Madrid, 28029 Spain; 6 Pathology Department, Hospital Universitario de Guadalajara, Guadalajara, 19002 Spain; 7 Experimental Therapeutics Group, VHIO - Vall d’Hebron Institute of Oncology, Barcelona, 08035 Spain; 8 Proteomics Unit, CNIO – Spanish National Cancer Research Center, Madrid, 28029 Spain; 9 Bioinformatics Unit, CNIO – Spanish National Cancer Research Center, Madrid, 28029 Spain; 10 Medical Oncology, Hospital Nacional Guillermo Almenara Irigoyen – ESSALUD, Lima, 15033 Peru; 11 Pathology Department, Hospital Universitario de Fuenlabrada, Fuenlabrada, 28942 Spain; 12 Medical Oncology, Hospital La Princesa, Madrid, 28006 Spain; 13 Genomics Unit, CNIC - Spanish National Center for Cardiovascular Research, Madrid, 28029 Spain; 14 Medical Oncology, Hospital Universitario Fuenlabrada, Madrid, 28942 Spain; 15 Medical Oncology, Hospital Universitario Quirón, Madrid, 28223 Spain

## Abstract

Triple-negative breast cancer (TNBC) lacks prognostic and predictive markers. Here, we use high-throughput phosphoproteomics to build a functional TNBC taxonomy. A cluster of 159 phosphosites is upregulated in relapsed cases of a training set (*n* = 34 patients), with 11 hyperactive kinases accounting for this phosphoprofile. A mass-spectrometry-to-immunohistochemistry translation step, assessing 2 independent validation sets, reveals 6 kinases with preserved independent prognostic value. The kinases split the validation set into two patterns: one without hyperactive kinases being associated with a >90% relapse-free rate, and the other one showing ≥1 hyperactive kinase and being associated with an up to 9.5-fold higher relapse risk. Each kinase pattern encompasses different mutational patterns, simplifying mutation-based taxonomy. Drug regimens designed based on these 6 kinases show promising antitumour activity in TNBC cell lines and patient-derived xenografts. In summary, the present study elucidates phosphosites and kinases implicated in TNBC and suggests a target-based clinical classification system for TNBC.

## Abstract (web summary)

Triple-negative breast cancer (TNBC) lacks prognostic and predictive markers. Here, the authors use phosphoproteomics to define kinases with distinct activity profiles in TNBC, demonstrating their prognostic value as well as their utility for simplifying TNBC classification and designing drug regimens.

## Introduction

Triple-negative breast cancer (TNBC) is an immunohistochemically defined breast cancer subtype that is negative for ER, PR, and HER2 expression, and has a poor prognosis^1^. A prognostic stratification system directly linked to different therapeutic options would be of great interest for TNBC. Several gene-centered approaches, such as high-throughput -omic studies assessing different properties of genes (NGS, CGH-arrays, methylomics, transcriptomics), have revealed a marked heterogeneity within TNBC^2–5^, with sets of mutations unique to individual patients^4^. In several cancer types, NGS studies have defined subtypes by point mutations, which are actionable targets in a number of malignancies, although not in breast cancer^6^. Highly penetrant oncogenes are rarely found in TNBC, suggesting that the TNBC phenotype is a result of coexisting, moderately penetrant, genetic changes that together contribute to its clinical presentation.

An alternative investigative approach is in-depth high-throughput phosphoproteomics, which exhibits certain advantages due to the fact that proteins are effector molecules in tumor cells^7–10^ and their functional status is modulated through post-translational modifications (PTMs), of which phosphorylation is the most ubiquitous during cellular signaling events^7–10^. These upper-level regulatory events are not covered by the assessment of gene-centric layers^11,12^. Previous studies in this area of research have preliminarily assessed the kinome in breast cancer at the gene expression level^13,14^. We hypothesized that the existing (genomic, transcriptomic) aberrations among different patients may coalesce into a discrete number of phosphorylation-driven patterns of activation of the proteome, the activity of which would determine the prognosis of a TNBC patient. These patterns would be driven by the activity of a certain number of kinases among the ~500 encoded by the human genome, many of which are currently targetable.

The aims of the present study were: (1) to define the phosphoprofiles differentiating the relapsed from the non-relapsed TNBC cases in a training set; (2) to identify novel phosphosites involved in TNBC biology and the identities of the hyperactive kinases driving the phosphoprofiles; (3) to evaluate the prognostic roles of these kinases in independent TNBC training sets; and (4) to preliminarily assess the therapeutic effect of blocking the kinases that drive the aggressive phosphoprofiles in preclinical models of TNBC. As a post-hoc analysis, the confluence of mutational patterns into kinase-activation patterns was investigated.

We found that a set of six kinases drives the phosphoprofiles of relapsing TNBC. These kinases preserve prognostic impact in two independent validation sets. When patients are classified in patterns according to the status of activation of these six kinases, two main patterns of activation with 44 sub-patterns can be found. Interestingly, several different genomic landscapes converge into each of these patterns. Finally, therapeutic studies in preclinical models suggest potential therapeutic utility of targeting these kinases in TNBC.

## Results

### Kinases that drive the phosphoprofile of the relapsed cases

**Fig. 1:**
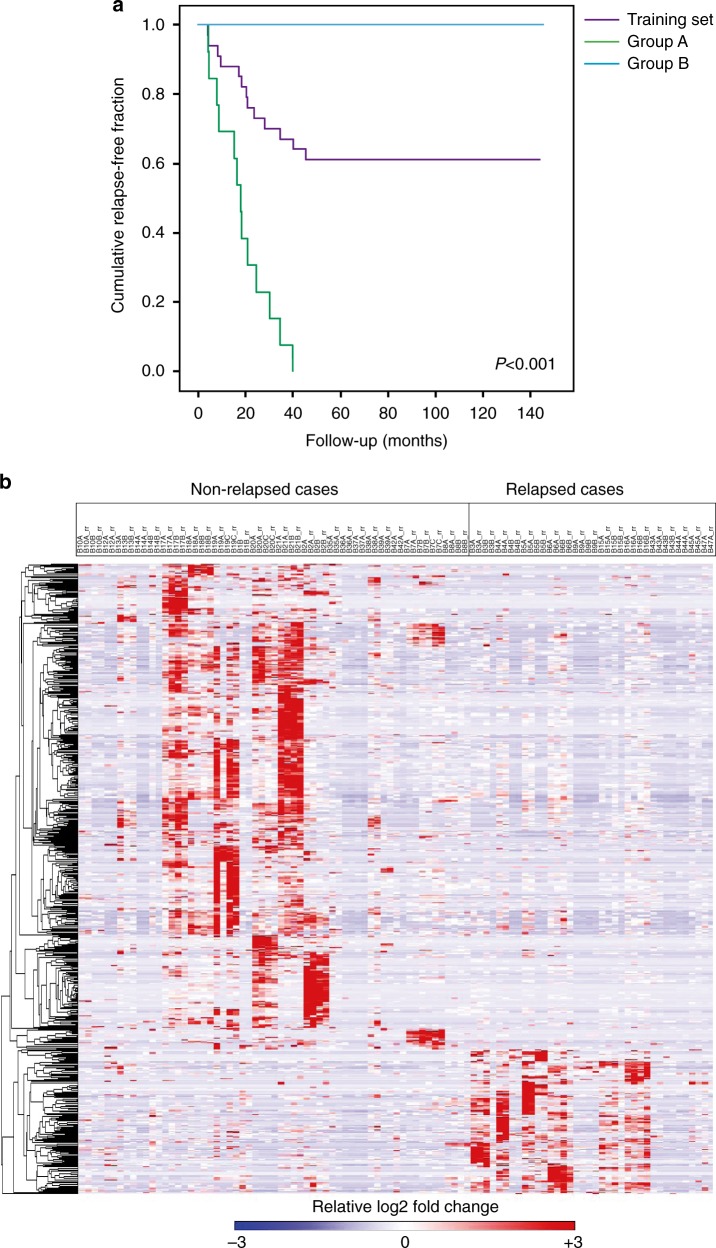
Phosphoprofiling of the patient training set. **a** There were two patient subgroups in the training set: A 13 patients who relapsed within <3 years after locoregional treatment (green chart), and B 21 patients who did not relapse >10 years after locoregional treatment (blue chart). The graphic shows the Kaplan–Meier curves of the training set as a whole (purple chart) and of the two subgroups. Median time to relapse in group A: 17.4 months; median time to relapse in group B: not reached (log-rank test, *p* < 0.001). **b** The heatmap shows the phosphopeptides (*n* = 702: 543 upregulated in non-relapsed and 159 upregulated in relapsed patients) with significant differences in phosphorylation levels in tumors from the training set by relapse status

The training set consisted of 34 tumor samples from patients with extreme phenotypes^15^ of TNBC. To maximize the chances of capturing the underlying biological factors accounting for the differences between the non-relapsed and relapsed phenotypes, we selected patients who relapsed in the first 3 years of follow-up after locoregional treatment and those free of relapse after 12 or more years, matched by conventional prognostic factors such as T, N, or G (Fig. 1a; Supplementary Table 1). Accordingly, we expected to have an increased chance of detecting phosphopeptides and kinases that could explain adverse outcomes. Phosphofractions of tumor samples purified by Ti(IV)-IMAC^16^ were analyzed by high-resolution, accurate-mass, tandem mass spectrometry (MS/MS), which yielded ~1.5 million spectra. We identified >10,000 nonredundant phosphorylation sites in >9000 phosphopeptides that mapped to at least 2643 distinct proteins (Supplementary Table 2).

**Fig. 2:**
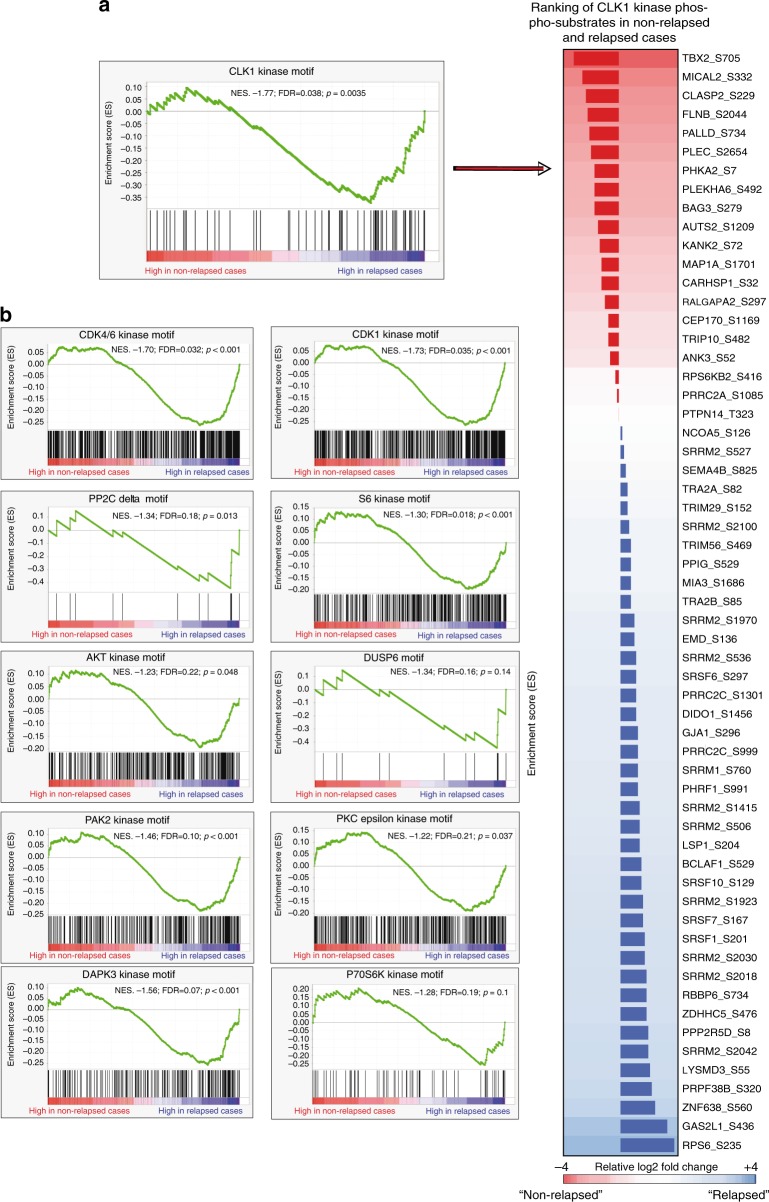
Kinases driving the profiles of the relapsed cases. **a** Example of a chart of normalized enrichment scores (NESs) (left) obtained for CLK1 from the relative abundance of its phosphorylated substrates in either the relapsed or the non-relapsed cases (right)—or kinase set enrichment analysis (KSEAS). Each substrate (phosphopeptide) is represented in the KSEA as a vertical black line. The proteins to which they map are represented in the right column by their encoding genes, adjacent to the site at which phosphorylation was detected. In this column, a larger or shorter horizontal bar depicts, for each substrate, the Log_2_-fold regulation in the relapsed (blue) versus non-relapsed (red) cases. **b** Two phosphatase (DUSP6 and PP2C-δ) and 9 kinase domains were enriched in the relapsed cases. The finding of an enriched phosphatase domain can be accounted for by the presence of a high concentration of a substrate for that phosphatase in a specific subgroup of patient tumors or cell lines. The in silico tool cannot predict whether a phosphatase is functional based on the absence of phosphorylation of its putative substrates; however, it can predict which upstream kinases or phosphatases can bind (and phosphorylate or cleave) an identified substrate. *P*-values and false discovery rates (FDR) are depicted for each kinase or phosphatase. Although most KSEAS show a low FDR, a relaxed FDR boundary (up to 0.25) was allowed to ensure as little information loss as possible in the mass spectrometry-to-immunohistochemistry translation step

We extracted functional readouts from the phosphorylation status of the peptides and translated this large data matrix to practical information. We first determined the abundance and nature of the phosphorylated peptides that distinguished relapsed from non-relapsed cases. The heatmap in Fig. 1b shows the 702 phosphopeptides identified in the tumors that demonstrated significant differential regulation; 159 peptides had significantly increased phosphorylation (at a higher intensity and frequency) in the relapsed versus the non-relapsed cases. Supplementary Table 3 shows the mapped proteins, the phosphorylated residue(s), the kinases predicted to elicit their phosphorylated status, the absolute intensity (abundance), and the percentage of relapsed and non-relapsed patients in whom these 159 peptides were detected. Sixty-three of these phosphosites have not previously been shown to be related to breast cancer. Moreover, since information regarding the function of many of the identified phopshosites is lacking, we sought to piggyback the phosphoprofiles onto their driver kinases, based on the nature and abundance of phosphorylation events and theoretical affinity of the consensus sequences for each kinase. We employed a methodology similar to that used for gene set enrichment analysis of gene expression data to identify clusters of interest^17^, and applied annotations to kinase motifs extracted using the Perseus software, which integrates 327 linear motifs (80 of which are SH2-binding domain motifs, 23 are phosphatase substrate motifs, and 224 are kinases motifs). This strategy of kinase set enrichment analysis (KSEAS) was used for the PSMs identified in the tumors, generating enrichment plots for the kinases that drive the profiles of relapsed versus non-relapsed cases. Figure 2a shows an example chart for the kinase, CLK1, for which increased activity was predicted in the relapsed cases based on the abundance/occurrence of its putatively identified substrates (right panel). The 11 KSEAS obtained from the phosphoprofiles of the relapsed cases are shown in Fig. 2a, b. Random substrates from the KSEAS predictions were tested with in vitro kinase assays coupled with mass spectrometry and proven to be real substrates of their predicted upstream kinases (Supplementary Figure 1). These data implicate several hyperactive kinases (CDK4/6, CLK1, CDK1, PP2Cδ, S6K, DAPK3, AKT, DUSP6, P70S6K, PAK2, and PKCε) in the relapsing TNBC phenotype. With the exception of the well-studied AKT kinase and to a lesser extent CDK4/6^18^, CDC2/CDK1^19^, PKCε^20^, S6K^13^, and CHK1^21^, these identified kinases have not yet been linked to aggressive TNBC. Therefore, we subsequently validated this information in an independent patient series.

### Translation of mass spectrometry to immunohistochemistry

We aimed to translate the information that we obtained by mass spectrometry (activated kinases—Fig. 2—and upregulated phosphosites—Supplementary Data 1) into data that could be obtained from FFPE samples (the common vehicle of tumor samples in the daily routine of the clinical environment), while seeking external validation of our results. Mass spectrometry-to-immunohistochemistry translation has 2 main challenges: (1) unknown criteria for defining an activated kinase and identifying the activated form in FFPE samples; and (2) lack of suitable reagents: currently, antibodies suitable for use in the immunohistochemical detection of most of the kinases or upregulated phosphosites identified by phosphoproteomics are not yet available. Supplementary Figure 2 explains the algorithm used to select the antibodies employed for external validation of the functional phosphosites depicted in Fig. 2 and Supplementary Data 1. The algorithm yielded 11 valid antibodies against the following targets: CDK6, CLK1, p-S6K (Ser^240^), DAPK3, PRKCE, p-AKT (Ser^473^), p-ERK (Thr^202^/Tyr^204^), p-P70S6K (Thr^389^), p-PNKP (Ser^114^/Thr^118^), CHK1, and c-Kit (control stainings are shown in Supplementary Figure 3).

### External validation

**Fig. 3:**
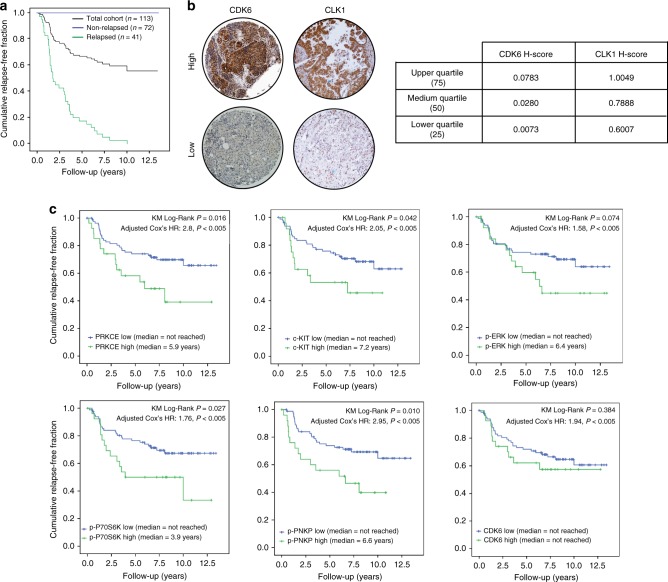
Independent validation. **a** Kaplan–Meier survival curve of the 113 patients comprising the first independent validation set—Val-1 (black chart); 72 of these patients did not relapse during 12.5 + years of observation (blue chart), whereas 41 patients did relapse (green chart). **b** Immunohistochemically stained examples of CDK6 and CLK1 from patients with H-scores in the upper (high) or lower (low) quartiles. **c** Kaplan–Meier curves according to the status (H-scores in the upper quartiles [green charts] or lower quartiles [blue charts]) of PRKCE, c-Kit, pERK (Thr^202^/Tyr^204^), pP70S6K (Thr^389^), pPNKP (Ser^114^/Thr^118^), and CDK6. The upper *p*-values shown in each chart were derived from the log-rank test comparing median overall relapse-free survival times (KM Log-Rank *p* value) between patients in the upper quartiles versus the lower quartiles. The hazard ratios depicted below correspond to those for relapses attributed to each kinase in a Cox model adjusted by T, N, G, and age, and are all statistically significant (Cox *P* *<* 0.005)

To ensure sufficient follow-up for the observation of all events in the series and coverage of all disease stages and treatment options, we gathered an independent set of 113 TNBCs, consecutively diagnosed in the year 2003, for validation purposes (Fig. 3a; Supplementary Table 1; Val-1). The distribution of H-scores among the quartiles and photomicrographs of stained tissue samples are shown in Fig. 3b; Supplementary Table 3 and Supplementary Figure 4, respectively. A kinase of interest with an H-score in the upper quartile was considered highly active. Six of the 11 kinases exhibited independent predictive power in Val-1, as shown by the Kaplan–Meier curves and/or Cox’s adjusted hazard ratios for patients with highly active kinases versus the remaining, according to their kinase H-scores (Fig. 3c: PRKCE, c-Kit, p-ERK (Thr^202^/Tyr^204^), p-P70S6K (Thr^389^), p-PNKP (Ser^114^/Thr^118^), and CDK6). The other 5 kinases showed no significant prognostic power (Supplementary Figure 5).

### Comparing kinomic and mutational landscape of TNBC patients

**Fig. 4:**
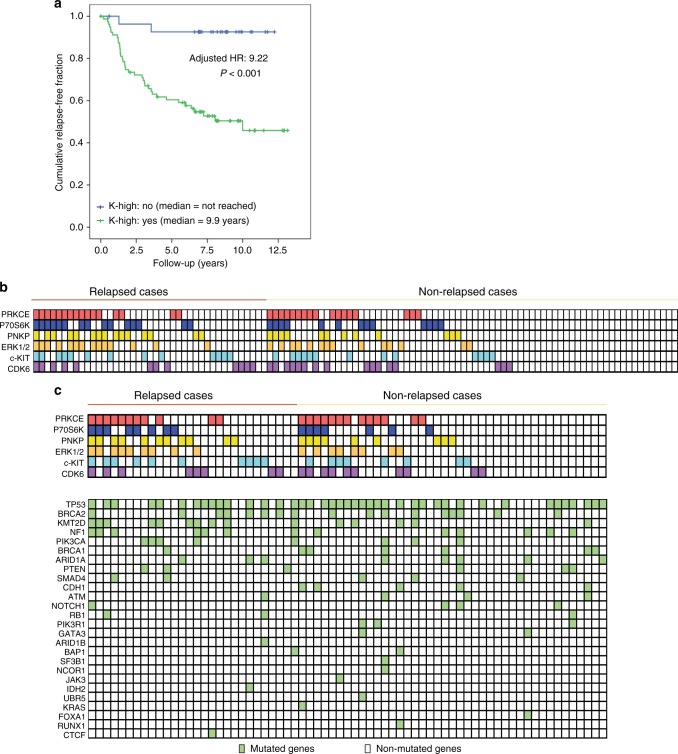
Clinical implications of kinomic and genomic landscapes. **a** Prognostic impact of the presence of one or more activated kinases**:** Kaplan–Meier curve for relapse of patients in validation set Val-1, whose tumors showed high activity of any of the 6 kinases (green chart: K-high = yes) versus the remaining (patients who showed low activity of the 6 kinases; blue chart: K-high = no). **b** Each of the 6 kinases with prognostic power is listed on the left side (rows), and for each patient (columns, stratified according to relapse status), the number of kinases with high-quartile staining are shown. **c** Below the kinase grid, a second grid contains the mutational status (yes/no, according to the filters explained in the Methods section) of the 25 most frequently mutated genes for each sequenced patient. The data show the “collapse” of mutational patterns into kinomic patterns; for instance, pattern 38 (Supplementary Table 5) was observed in patients both with and without TP53, BRCA2, PTEN, or ARID1A mutations. The same statement was also valid for other frequently observed kinase patterns in the validation set, such as c-Kit-high (pattern 36 (*N* = 9 patients), which accounted for patients with various combinations of wild-type and mutant TP53, BRCA2, PIK3CA, and PTEN) or CDK6-high

Given the lack of co-linearity among the H-scores of the different kinases (Supplementary Figure 6), and the lack of known direct functional interplay, we expected none of the kinases to be necessary for relapse but to perhaps independently contribute to the relapsed phenotype. We analyzed the outcomes of patients whose tumors showed high activity of any of the 6 kinases versus patients whose tumors showed no such activity. The resulting variable (K-high) allowed stratification of the 113 patients into two groups; one containing 81 cases (71%, high activity of any of the 6 kinases) and one containing 32 cases (29%, low activity of any of the 6 kinases). The respective relapse rates were 47% and 6.5% after 12 + years follow-up, and the respective median times to relapse were 9 years and not reached (Log Rank *P* *<* 0.001; Fig. 4). Following adjustments for T, N, G, and age, K-high positivity showed a Cox’s hazard ratio for relapse of 9.22 (Cox *P* < 0.001), suggesting that the group of patients without high activity of any of the 6 kinases defines a subgroup of TNBCs with an exceptionally good prognosis, and conversely, K-high defines a subgroup with an extremely poor prognosis. For test purposes, different variables were built combining the values of only 2, 3, 4, or 5 kinases; however, despite showing better prognostic discrimination than any of the kinases alone, none achieved the same prognostic accuracy as K-high (Supplementary Figure 7).

The number of activated kinases per patient varied substantially; nevertheless, out of 64 possible patterns, we found only 38 in Val-1 (Fig. 4b, Supplementary Data 2). In total 12 patterns were exclusive to the relapsed cases. The most frequent pattern was the absence of activation of any of the 6 kinases, which was associated with a >93% rate of relapse-free survival at 12.5 years. The more commonly detected patterns were those showing activation of a single kinase (p-P70S6K, CDK6, or c-Kit), although most patterns involved diverse combinations of kinases. Interestingly, no trends were observed in patterns showing mutually exclusive or co-occurring kinases, which is in contrast to gene mutation patterns^4–6,22,23^. These results suggest that different gene mutation patterns may converge to activate each of these kinases, in turn, driving tumor progression. When comparing the overlay of the mutational status of the top 25 most-frequently mutated genes in breast cancer and the kinase classification, it can be appreciated that each kinase activation pattern could be achieved by different mutational landscapes (Fig. 4c). These data highlight the importance of a kinase-based classification as compared with one based on genes.

Patients from Val-1 were diagnosed more than a decade ago, and treatment standards have since changed. The current standard for patients with tumors >1 cm includes a combination of one anthracycline, cyclophosphamide, and one taxane (A + C + T). A + C + T was only administered to certain patients (*n* = 27; 25%) from Val-1 who showed adverse classic prognostic factors; thus, we gathered a second validation set (Val-2), diagnosed from 2009, of 61 TNBC patients that received A + C + T (Supplementary Table 1). Val-2 served two purposes: (1) to validate K-high, which was built with 6 out of 11 candidate kinases from the training set; and (2) to test the role of K-high in a modern series. Despite the fact that Val-2 had a shorter follow-up period, and therefore a smaller proportion of patients experienced relapse, K-high was still highly significant (HR: 4.47; Cox *P* = 0.029; Supplementary Figure 8A). The kinase-pattern distribution across this series was similar to that of Val-1, although 6 new patterns emerged from this series (Supplementary Figure 8B, Supplementary Data 3).

### Targeting activated kinases in preclinical models

Various analytical approaches have been used to narrow-down drug candidates for use in the design of drug regimens on the basis of different -omic data^24–27^. With the aim of exploring a less laborious approach, we tested a very simple alternative; to determine the levels of each of the 6 active kinases, whose hyper-function appeared to drive the fate of aggressive cases, and select combined agents of interest according to the levels of these targets. To this end, several TNBC cell lines (in vivo characterization, phosphoprofiling, and KSEAS are shown in Supplementary Figures 9 and 10, and Supplementary Table 2) and patient-derived xenografts were chosen for further study.

**Fig. 5:**
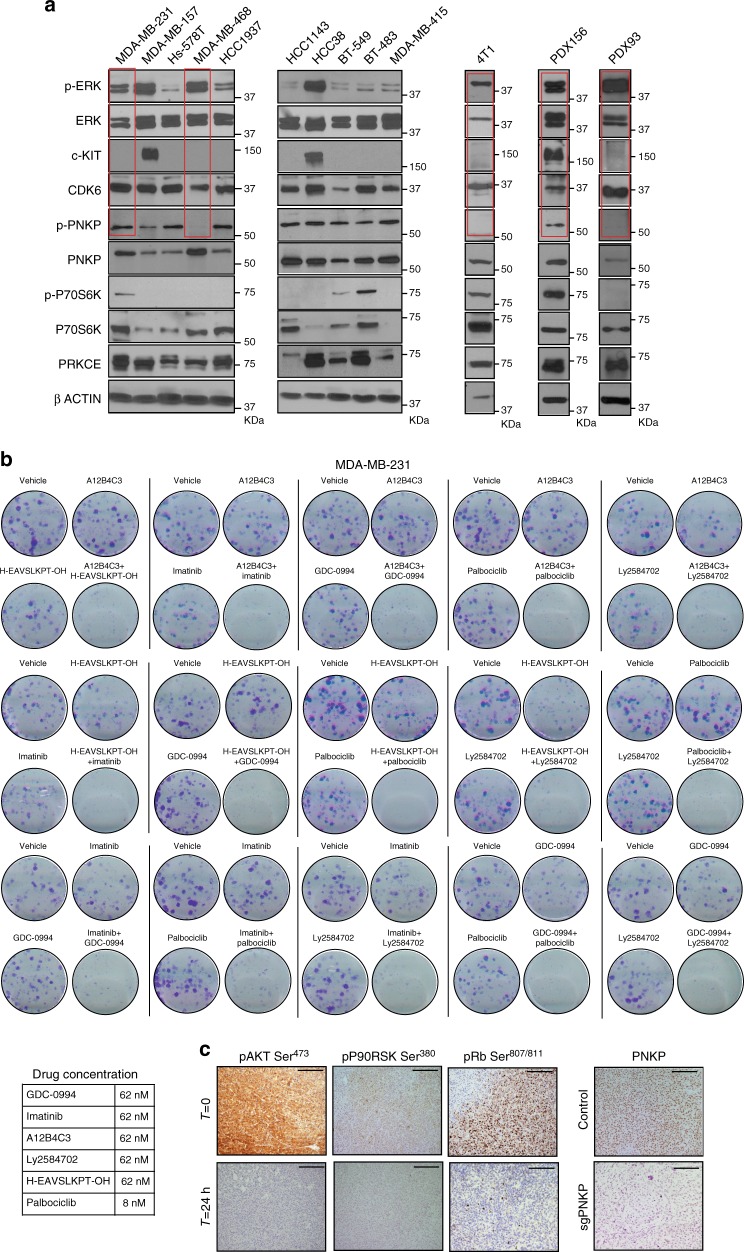
In vitro therapeutic efficacy and in vivo pharmacodynamics. **a** Western blotting showing the levels of each of the 6 kinases in the signature, in addition to their non-phosphorylated controls where applicable, in 10 human TNBC cell lines, the transplantable murine TNBC tumor model 4T1, and 2 patient-derived xenografts (PDXs). The three targets against which clinical-grade drugs are available are highlighted in red. **b** Colony assays (MDA-MB-231) showing the differences between single-target versus two-target pharmacological blockade. For each of the 15 possible 2-by-2 drug combinations using the 6 agents against the kinases in K-high, a representative vehicle-treated well, representative single-agent-treated wells and a well containing the doublet are shown. All 4-well images belong to unique 12-well dishes. Representative images of three independent experiments. **c** In vivo dosage of imatinib, GDC-0994, and palbociclib at standard doses for animal use led to decreased AKT, P90RSK, and RB phosphorylation levels (targets of the kinases c-Kit, ERK, and CDK6, respectively) in MDA-MB-231-xenografted tumors after 24 h. The right panel shows total PNKP levels in wild-type (upper) and CRISPR PNKP MDA-MB-231 transfectant (lower panels) tumors. Scale bar, 50 μm

The levels of PRKCE, c-Kit, p-ERK (Thr^202^/Tyr^204^), p-P70S6K (Thr^389^), p-PNKP (Ser^114^/Thr^118^), and CDK6 for each preclinical model are shown in Fig. 5a. Three clinical-grade (palbociclib, imatinib, and GDC-0994; which target CDK6^28^, c-Kit^29^, and ERK^30^, respectively) and three non-clinical-grade (A12B4C3, the peptide H-EAVSLKPT-OH. and Ly2584702; which target PNKP^31^, PRKCE^32^, and P70S6K^33^, respectively) agents were tested. To the best of our knowledge, there are no clinical-grade agents available against the latter kinases. The 6 agents demonstrated limited single-agent in vitro activity (Fig. 5b, Supplementary Figures 11 and 12). Since the kinases tended to be non-colinear (Supplementary Figure 6), and many TNBC cases harbored activation of more than one kinase (Fig. 4), taken together these data suggest that no particular kinase is essential for TNBC progression and that several can co-exist within each individual tumor, contributing to the aggressive TNBC phenotype. Thus, combining the clinical-grade agents two-by-two seemed like a reasonable approach. Indeed, the combination of low-activity or even virtually inactive single-agent doses into doublets yielded profound inhibitory effects in MDA-MB-231 (the most aggressive of these cell lines; Supplementary Figure 9), Hs-578T, and MDA-MB-468 (Fig. 5b, Supplementary Figures 11 and 12) colony assays.

**Fig. 6:**
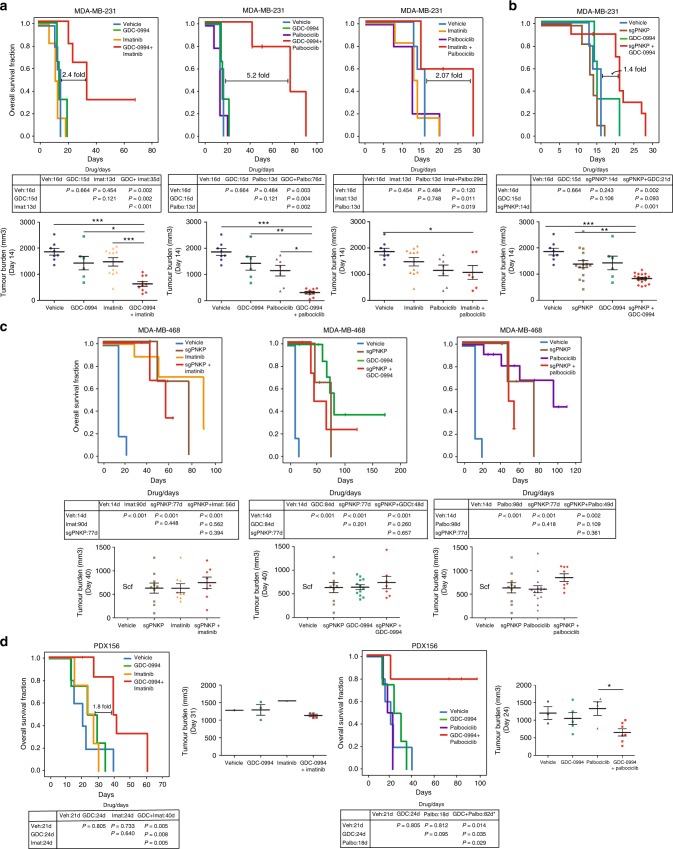
In vivo therapeutic efficacy. **a** MDA-MB-231 is a cell line with high levels of p-ERK and CDK6, but no visible levels of c-Kit. It can be observed that, although imatinib-based doublets significantly prolonged mice overall survival as compared with the singlets or vehicle, the magnitude of the improvement is much lower than that seen with the p-ERK + CDK6-targeting doublet (GDC-0994 and palbociclib, mid chart; >5-fold increase in overall survival compared with ~2-fold). Below the Kaplan-Meier curves, the median overall survival (days) for each combination (or single-agent) in addition to the Log-Rank *P*-values are shown. Finally, representative tumor burdens of each treatment group are depicted. **b** MDA-MB-231 shows relatively high p-PNKP levels; CRISPR PNKP MDA-MB-231 transfectant xenografts treated with GDC-0994 also showed statistically significantly longer overall survival as compared with GDC-0994 administered to wild-type MDA-MB-231 or untreated CRISPR PNKP transfectants. Mice treated with: vehicle (*n* = 5), imatinib (*n* = 6), GDC-0994 (*n* = 4), palbociclib (*n* = 5), GDC-0994 + palbociclib (*n* = 5), palbociclib + imatinib (*n* = 5), sgPNKP (*n* = 11) and sgPNKP + GDC-0994 (*n* = 11). **c** Compared to MDA-MB-231, the levels of p-PNKP in MDA-MB-468 are almost undetectable. The levels of p-ERK and CDK6 are high, and those of c-Kit are low. Matching the observations in the other models, when a targeted doublet includes a target with low or absent expression (namely, p-PNKP in MDA-MB-468), no synergy is observed. In the three Kaplan-Meier curves it can be observed than the doublet is not better than any of the monotherapies (or sgPNKP alone). Representative tumor burden charts are shown below the survival curves. Mice treated with: vehicle (*n* = 6), sgPNKP and sgPNKP + imatinib (*n* = 5), imatinib (*n* = 8), GDC-0994 (*n* = 11), GDC-0994 + imatinib (*n* = 13), palbociclib (*n* = 12), GDC-0994 + palbociclib (*n* = 9), sgPNKP + palbociclib (*n* = 4) and sgPNKP + GDC-0994 (*n* = 7). **d** Finally, in PDX156 (c-Kit and pERK higher than CDK6), imatinib plus GDC-0994, and GDC-0994 plus palbociclib significantly prolonged median overall survival compared to the monotherapies. Mice treated with: vehicle (*n* = 5), imatinib (*n* = 4), GDC-0994 (*n* = 4), palbociclib (*n* = 4), GDC-0994 + palbociclib (*n* = 5) and GDC-0994 + imatinib (*n* = 9). In tumor burden graph, each point represents a tumor. The data are represented as mean±SEM and Student ´s *t* test was performed. *p < 0.05, **p < 0.01, ***p < 0.001

Further, in vivo models showed important effects of drug combinations selected by simple assessment of the target levels. Clinical-grade agents were tested in doublets to assess the efficacy and selectivity of the approach. MDA-MB-231 showed high levels of pERK and CDK6, together with almost negligible levels of c-Kit (Fig. 5a). Palbociclib, GDC-0994, and imatinib were combined in pairs at the recommended doses for preclinical studies^28–30^, showing proof-of-pharmacodynamic effect on their targets (inhibition of p-AKT-S473^34^, p-P90RSK-S380, and p-Rb-S807/811^28^) (Fig. 5c). Kaplan–Meier curves and median overall survival of MDA-MB-231-grafted animals treated with the three drug combinations are shown in Fig. 6a, in addition to tumor burden comparisons, showing a more profound effect for palbociclib plus GDC-0994 than for the imatinib-containing combinations. High levels of p-PNKP were found as well in several cell lines. Given the absence of effective compounds, we tested the effect of PNKP deletion by CRISPR in MDA-MB-231 (high p-PNKP) and MDA-MB-468 (virtually absent p-PNKP). Intratumour deletion of PNKP is shown in Fig. 5c. Again, only statistically significant advantages were obtained for the combination targeting ERK and PNKP in MDA-MB-231 (Fig. 6b), but not in the model with low p-PNKP (Fig. 6c). Similarly, the 4T1 murine TNBC transplantable model, showed high levels of p-ERK and CDK6 but no detectable levels of c-Kit (Fig. 5a). The combination of palbociclib plus GDC-0994 led to a statistically significant improvement in overall survival, as opposed to the combination of imatinib plus palbociclib (Supplementary Figure 13A). Finally, patient-derived xenografts (PDXs) are considered better mimics of human tumors than cell line xenografts for drug development. Two PDX (one with high c-Kit, p-ERK, and CDK6 activity—PDX156—and one with high p-ERK and CDK6 activity only—PDX93) were tested (Fig. 5a). Figure 6d shows that both imatinib plus GDC-0994 and palbociclib plus GDC-0994 improved overall survival compared to each treatment alone. However, in PDX93, only palbociclib plus GDC-0994, but not the imatinib-based doublets, achieved statistically significant differences in tumor control (Supplementary Figure 13B). Taken together, these data suggest that the levels of the kinases in K-high, which drive aggressive TNBC biology, appear to aid selection of active and relatively specific doublets in TNBC preclinical models.

## Discussion

While gene-centered approaches have brought unprecedented knowledge to breast cancer drivers and subtypes^3,4,13,22,23,35,36^, in the case of TNBC, this novel information has revealed extreme heterogeneity that has, to date, precluded a definition of prognostic/predictive factors. The lack of oncogenic addiction drivers in TNBC renders conventional chemotherapy the main treatment option.

Previous studies have carried out the initial steps of pinpointing the main kinases involved in breast cancer^13,14,37^; however, mass spectrometry of TiO2-IMAC-purified phospho-fractions can achieve a greater depth of phosphoproteome coverage, with very large dynamic ranges and high specificity compared with other alternatives^38–40^. In addition, the KSEAS methodology allows the estimation of a functional readout from the spectra, allowing summarization of large phosphoprofiles into their driving kinases. Two previous studies have implemented the KSEAS methodology while portraiting the main aberrant signaling axes in prostate^41^ and ovarian cancers^42^; however, these studies did not link this information to clinical outcome. Another study combining mass spectrometry assessment of the proteome with a multi-layer -omic interrogation of breast cancer allowed the discovery of the paths linking genomic to signaling aberrations^43^. Here, we aimed to advance the field of TNBC taxonomy and show proof-of-concept of a kinase-based classification of TNBC linked to clinical outcomes.

Several findings of the present study deserve attention. First, the reduction of the initial >10,000 phosphosites identified by mass spectrometry to a selected number of TNBC-driver kinases allowed the translation of a large amount of data, revealing an upper level of regulation by a few elements; that is, we identified TNBC clusters defined by the activity of a few kinases (functional clusters) (Figs. 2, 4, and Supplementary Figure 8B; Supplementary data 2 and 3). Six kinases with increased activity that appeared to drive the aggressive clinical course of TNBC were identified (Figs. 3, 4 and Supplementary Figure 8A). Investigations regarding the potential prognostic, predictive, or therapeutic role in TNBC of the majority of these kinases (with the exception of ERK) are yet very preliminary^44–46^ or absent. Together with the identification of 63 novel phosphosites in the phosphoprofile of the relapsed cases (Supplementary Data 1), this information lays the foundations for further basic research on signaling axes driving the aggressive biology of TNBC and indicates that our strategy is valuable as a discovery tool.

Moreover, the kinase hits extracted from the present study allowed the construction of a basic kinomic landscape of TNBC according to the kinase status (high or low activity), which was linked to disease course. When considered together, the status of the 6 kinases was more informative than any separately. According to the status of the 6 kinases, we found two main TNBC patterns; of which, one, without high activity among any of the 6 kinases, was associated with a long-term relapse-free rate of 93%. The second pattern (with 37 sub-patterns) was characterized by the hyperactivity of one or more kinases, with all but two of the patients who relapsed having tumors that showed this pattern (Fig. 4, Supplementary Data 2). Belonging to any of these 37 sub-patterns conferred a greater than 9-fold risk of relapse, even when adjusted for the conventional risk factors. Similar results were obtained with a second validation set (Supplementary Figure 8 and Supplementary Data 3). To the best of our knowledge, there is no other prognostic factor or signature that can stratify the outcomes of patients with TNBC with the accuracy of K-high. The ability to identify patients belonging to this subgroup is useful from a clinical point of view, since therapeutic efforts can be focused on those patients who are not in this group. Future studies should compare the prognostic performance of this approach and gene expression-based classifications^2^.

Furthermore, a key taxonomic question that was formulated following the observation that a relatively parsimonious classification could be obtained based on phosphoproteomic analysis, is whether complex genomic mutation patterns can be condensed into simpler classification systems. TNBC taxonomic studies have not yet revealed associations between the mutational status of single genes or combinations of several genes and disease outcomes^4,5,36^. The possibility of kinase patterns encompassing complex genomic patterns was assessed in the present study in a post-hoc manner; thus, several samples could not be sequenced due to insufficient or inadequate leftover tissue, and this constitutes a study limitation. However, we believe that the data shown in Fig. 5 serve as proof-of-concept of the fact that different genetic landscapes converge into the same kinase-activation pattern. Thus, diseases with complex genomic landscapes, such as TNBC, may benefit from kinase-based classifications.

Finally, the kinase-based classification shows that several signaling axes have high activity in patients that relapse despite adequate treatment according to current standards. This fact may have future therapeutic implications. Kinase pattern-oriented treatments may simplify current precision medicine approaches that aim to “treat genes”, since: (1) most known mutations involved in TNBC are not oncogenic addiction drivers; (2) several mutations with complex functional implications exist at the individual tumor level; (3) different mutations converge into a given kinase-activation pattern; and (4) most kinases in the signature are, or soon will be, targetable with compounds under current development. We have provided preliminary experimental evidence suggesting that a simple assessment of activated kinases could guide the design of tailored regimens. The data shown in Fig. 6 and Supplementary Figure 13 suggest a certain degree of specificity of the approach. Nevertheless, the detected efficacy of imatinib in models with little c-Kit levels suggest either certain off-target effect of the agent or, most likely, that the specificity is not 100%. Different analytical approaches that integrate high-throughput data have suggested that powerful drug-combinations can be designed on the basis of multi-omic data^24–27^. Although the objective of this preliminary therapeutic approach is not to compare its performance to the former, it seems quite simple and non-time-consuming. Future mechanistic insights into the manner by which the activity of the 6 kinases drives TNBC progression (role in therapeutic resistance, metastasis, or other aggressive features) are necessary to ascertain how and when to use clinical grade inhibitors. Pilot proof-of-concept trials studying the aggregation of efficacy of doublets according to the status of the 6 kinases should establish the role of this approach in a clinical setting. It is important to highlight, however, the positive effects found for palbociclib or imatinib when used in combinations guided by the present taxonomic findings; since, previous preclinical studies have suggested a limited role for CDK4/6 inhibition in TNBC^47^, and clinical studies using imatinib alone in unselected^48^ or c-Kit-positive breast cancer patients has also shown limited efficacy^49^.

The present study has certain limitations. First, the accuracy of the classification may be compromised in the mass spectrometry-to-immunohistochemistry step. Second, since current kinase enrichment predictions are in silico tools based on available kinase-substrate knowledge, when databases are updated with novel data regarding kinase catalytic affinity and substrate identification, novel predictions may emerge from the present data. This may explain, for instance, why a PIM1 kinase enrichment (a kinase recently linked experimentally to TNBC^50,51^) was not found in the present data. Third, while translation to immunohistochemistry appears to be a necessary step to gain widespread use of this approach, sample handling, staining protocols, and pathologist evaluations may vary considerably among hospitals, and standardizations in this regard will be necessary in the future. Tissue hypoxia and fixation times can bias the pathway enrichment found in the analysis of the captured phosphosites (increasing the detection of phosphosites related to stress, cell death, and transcriptional regulation pathways^52^) and decrease the sensitivity of mass spectrometry for phospho-detection^53,54^, respectively. Despite the fact that the country biobank standard operational procedures reinforce standardization of maximum hypoxia (20 min) and fixation (24 h) times, and we did not find significant enrichment in those pathways, both constitute sources of variability in the technique and represent a weakness for widespread validation. Fourth the present validation studies were conducted in the adjuvant setting only. Future independent validations that test the role of K-high in other scenarios (neoadjuvant, metastatic) are warranted.

In summary, we have shown for that in vivo phosphoproteomics can be used to predict outcomes in TNBC, simplify its classification, and select treatment doublet candidates for clinical testing. Future studies and trials by independent groups should now find an adequate place for tumor phosphoproteomics in routine clinics, akin to the case of the seminal studies performed with gene expression almost 20 years ago.

## Methods

### Patient tumor samples

Samples from the training and validation set were gathered through the Spanish National Biobank Network. The study was conducted in accordance with the Declaration of Helsinki. Institutional review board approval was obtained at the Instituto de Salud Carlos III (study number: CEI PI 30-2010). Informed consent was obtained from all participants. None of the patients received neoadjuvant therapy prior to biopsy or were BRCA-1 deficient.

### Cell lines

A panel of human triple negative breast cancer cell lines (MDA-MB-231, MDA-MB-157, Hs578T, MDA-MB-468, HCC1937, HCC1143, HCC38, BT549, BT483, and MDA-MB-415) was acquired from the American Type Culture Collection (ATCC). Cells were maintained following the ATCC recommendations. Luminescent cell lines were generated by stable transfection with a plasmid encoding firefly luciferase (pGL.4.51 luciferase reporter vector, Promega). All cell lines were confirmed to be mycoplasma negative by Mycoalert^TM^ Mycoplasma Detection Kit (Lonza).

### Colony assays

Breast cancer cell lines MDA-MB-231, MDA-MB-468 and Hs-578T cell lines were seeded at a density of 200, 2000 and 2000 cells per well respectively in 12-well plates. After overnight incubation, drugs were added to the tissue-culture media. Media was replaced every 5 days. After 10 days of culture, cells were fixed and stained with 0.1% (w/v) crystal violet in 10% (v/v) ethanol. All experiments were performed in triplicate.

### Kinase assays

Kinase reactions were carried out at 30 °C for 30 min using specific kinases buffer. Recombinant P70 S6Kinase (1-421, TA412E, Millipore) was incubated with recombinant IRS2 (H8660-Q01, Abnova) or RPS6 (H6194-P01, Abnova) in kinase buffer (20 mM Tris–HCL pH 7.5, 20 mM MgCl_2_, 1 mM DTT, 1 mM EDTA, 1 mM sodium ortovanadate, 0.4 mM PMSF, 20 mM glicerphsphate) in the presence of 50 μM cold ATP and 1.5 uCi [32 P] ATP. CDK6/Cyclin D3 complexes (14-519, Millipore) in 10 mM MOPS/NaOH pH7, 1 mM EDTA, 0.1% B-mercaptoethanol, 10 mM Magnesium acetate were incubated with WEE1 (H7465-P01, Abnova), HSF1-11R (hTF-0070, LD Biopharma) or RUNX1 (hTF-0380, LD Biopharma) in the presence of 100 μM cold ATP and 2 uCi [32 P] ATP. Recombinant PKCE kinase (P2282, Thermofisher) was incubated with recombinant IRS2 or RPS6 in the following kinase buffer (60 mM HEPES-NaOH pH7.5, 3 mM MgCl2, 3 mM MnCl_2_, 1 mM CaCl_2_, 4 mM EDTA, 3 μM Na-orthovanadate, 5 μg/ml phosphatidilserine, 1 ug diacylglycerol, 1.2 mM DTT, 50 μg/ml PEG20000 in the presence of 50 μM cold ATP and 1.5 μCi [32 P] ATP. In all cases, reactions were stopped by addition of Laemmli sample buffer. Radioactive samples were subject to acrylamide gel electrophoresis, followed by gel drying and autoradiography.

The samples were then prepared for proteomic analysis as follows: proteins were in-gel digested with trypsin using the standard procedure. Briefly, bands were de-stained with 50 mM ammonium bicarbonate in 50% acetonitrile solution, reduced with 15 mM TCEP at 45 °C for 60 min and subsequently alkylated with 30 mM chloro-acetamide at RT for 45 min. Digestion was performed overnight with a trypsin solution (6.25 ng/mL) at 37 °C. Supernatant was collected and peptides were further extracted from the gel plugs with 5% TFA. Resulting peptides were desalted using home-made C18 stage-tips.

Finally, we detected the phosphorylated peptides by mass spectrometry. LC–MS/MS was done by coupling an UltiMate 3000 HPLC system to a Q Exactive Plus mass spectrometer (Thermo Fisher Scientific). Peptides were loaded into a trap column Acclaim™ PepMap™ 100 C18 LC Columns 5 µm, 20 mm length) for 3 min at a flow rate of 10 µl/min in 0.1% FA. Then peptides were transferred to an analytical column (PepMap RSLC C18 2 µm, 75 µm×50 cm) and separated using a 60 min effective curved gradient (buffer A: 0.1% FA; buffer B: 100% ACN, 0.1% FA) at a flow rate of 250 nL/min from 2 to 35% of buffer B. Peptides were electrosprayed (1.9 kV) using an EasySpray ion source (Thermo Fisher Scientific), a heated capillary (250 °C) and S-Lens RF level of 60%. The mass spectrometer was operated in a data-dependent mode, with an automatic switch between MS (350–1500 m/z) and MS/MS (fixed first mass 100 m/z) scans using a top 15 method and a dynamic exclusion of 25 s. MS and MS/MS spectra were acquired in the Orbitrap with a resolution of 70,000 and 17,500 FWHM (measured at 200 m/z) respectively. Peptides were isolated using a 2.0 Th window and fragmented using higher-energy collisional dissociation (HCD) with a normalized collision energy of 27. The ion target values were 3E6 for MS (25 ms max injection time) and 1E5 for MS/MS (45 ms max injection time). For very faint bands, maximum injection times were increased to 90 ms (AGC value = 5E4).

Raw files were processed with Proteome Discoverer 1.4 (Thermo Fisher Scientific) using Sequest HT against a human protein database (UniProtKB 20,558 sequences) supplemented with contaminants. Carbamidomethylation of cysteines was set as a fixed modification whereas oxidation of methionine and phosphorylation of serine, theronine and tyrosine as variable modifications. Minimal peptide length was set to 6 amino acids and a maximum of two tryptic missed-cleavages were allowed. Precursor tolerance was set to 10 ppm and fragment ion tolerance to 0.025 Da. Results were filtered at 0.01 FDR using Percolator. Abundances of phosphopeptides were calculated using the extracted ion chromatograms (with a 10 ppm window around the M + 0 isotope peak for 30 s before and after the peptide’s MS/MS). In addition, phosphopeptide abundances were normalized by total protein abundance, which was estimated based on non-phosphopeptides. To this end, raw files were also processed with MaxQuant 1.5.7.4 using the standard settings against a human protein database (UniProtKB 20,585 sequences) supplemented with contaminants. Label-free quantification was done with match between runs (match window of 0.7 min and alignment window of 20 min). Carbamidomethylation of cysteines was set as a fixed modification whereas oxidation of methionines, protein N-term acetylation and phosphorylation of serines, threonines and tyrosines as variable modifications. Minimal peptide length was set to 7 amino acids and a maximum of two tryptic missed-cleavages were allowed. Results were filtered at 0.01 FDR (peptide and protein level). Protein intensity values (obtained from the proteingroups.txt file) were then used to calculate the normalization factor needed in each case.

### Mouse models

All animal experiments were approved by the Institutio de Salud Carlos Tercero Ethics Committee (PROEX/027/15) and performed in accordance with the guidelines stated in the International Guiding Principles for Biomedical Research Involving Animals developed by the Council for International Organizations of Medical Sciences. Four- to 6-week-old female athymic nude mice (Hsd: Athymic Nude-Foxn1nu) were purchased from Charles River Laboratories. For the metastasis model, 5 × 10^6^ luciferase-expressing cancer cell lines were suspended in 200 μl of 1× PBS and injected intraperitoneally into female nude mice. Bioluminescence signal was followed once per week by IVIS Spectrum imaging system (PerkinElmer). For mammary fat pad injections 1 × 10^6^ cells were re-suspended in 50% Matrigel (Corning) and injected (50 μl volume). Regarding patient-derived xenografts (PDXs) Surgical or biopsy specimens from primary tumors or metastatic lesions were immediately implanted in mice. Fragments of 30 to 60 mm^3^ were implanted into the mammary fat pad (surgery samples) or the lower flank (metastatic samples) of 6-week-old female athymic HsdCpb:NMRI-Foxn1nu mice (Harlan Laboratories). Upon growth of the engrafted tumors, the model was perpetuated by serial transplantation onto the lower flank.

Tumor formation and growth were monitored weekly by using calipers. Tumor volumes were calculated using the formula *V* *=* (*D* °−*d*2)/2 mm^3^, where *D* is the largest diameter and *d* is the shortest diameter. Mice were euthanized when reaching humane end point (1500 mm^3^). Tumors were excised and fixed (10% formalin solution) for histological examination (FFPE) or snap-frozen for subsequent analysis.

### Animal treatments

Animals were randomized (http://www.randomization.com/) to receive vehicle, single agents or combinations when the tumors reached 500 mm^3^ size (intramammary tumors) or two days after the intraperitoneal injections. Sample size was chosen based on previous data for similar assays. For the animal treatments palbociclib, dissolved in 50 mM sodium lactate buffer, pH 4.0, was administered by oral gavage at 100 mg/kg/day. GDC-0994 was dissolved in 2% DMSO + 30% PEG-300 + 5% Tween80 + dH2O and administered by oral gavage at the dose of 50 mg/kg/day. Imatinib was dissolved in sterile saline and administered by intraperitoneal injection at the dose of 70 mg/kg/day. Investigators were blinded to treatment assignment during the experiment. Tumor growth inhibition (TGI) was calculated using the following formula: TGI = [1−(*T*F/*T*0)A/(*T*F/*T*0)V] × 100, where *T*F is the time point analyzed, *T*0 is the initial time, A () is the tumor measurement corresponding to drug treatment, and V() is the tumor measurement from the vehicle treatment. The same formula used for the in vivo experiments for antagonism, indifferent or additive activity threshold was used for TGI. The effects of the combinations were calculated at the last time point when the animals belonging to the treatment group that was first terminated because of tumor growth.

### Generation of PNKP knockout cells

Clustered Regularly Interspaced Short Palindromic Repeats (CRISPR)/Cas9 All-in One Lentivector system was purchased from Applied Biological Materials (ABM). Recombinant lentiviruses were generated in HEK 293T cells by cotransfection of sgRNA-encoding plasmids targeting human PNKP (Cat#K1676005) with pVSV-G and psPAX2 packaging plasmids (Addgene). MDA-MB-231 and MDA-MB-468 cells were infected with the sgRNA-encoding lentivirus and stable cells were established by puromycin selection (1.5 μg/ml) (Sigma). After 7 days of lentiviral infection, protein levels of PNKP were measured by western blot.

### Immunohistochemistry

Samples were included in fresh 10% neutral buffered formalin immediately after surgical excision. The lag times from excision to fixing and intra-hospital tissue mobilization procedures were minimized following the protocols proposed for tissue biobanking. Cold ischemia times varied from 30 s to a maximum of 20 min. Formalin-fixation times ranged from 16 to 24 h (from surgical excision until the next morning). Tissue microarrays were mounted with two 1-mm cores per sample (MTA-I, Beecher Instruments) with the validation set samples. An expert pathologist examined a template H&E slide from each sample to select the areas for core selection.

Immunohistochemical staining was performed on 5-μm TMA sections. Deparafinization and antigen retrieval (cell conditioning) were performed on DISCOVERY XT automated slide staining system using validated reagents (Ventana Medical Systems, Inc.).

The following antibodies were used for IHC: phospho-AKT (Ser473) (D9E, #4060) (1:10), phospho-p70S6K (Thr389) (1A5, #9206) (1:150), phospho-p44/42 (ERK1/2) (Thr202/Tyr204) (#9101) (1:100), phospho-S6 Ribosomal Protein (Ser240/Ser244) (D68F8, #5364) (1:250), phosho-PNKP (Ser114/Thr118) (#3522S) (1:50), phospho-p90RSK (Ser380) (D3H11, #11989S) (1:30) and phospho-Rb (Ser807/811) (D20B12, #8516) (1:100) (all from Cell Signaling Technology); CDK6 (98D/H8, Monoclonal Antibodies Core Unit, CNIO) (1:1); PKC Epsilon (EPR1482(2), #LS-B7689/36454) (1:200), DAPK3/ZIPK (#LS-B557/43125) (1:10) and phospho-CHK1 (Ser45) (#LS-C177888) (1:50) from LifeSpan BioScience; PIM1 (12H8 #sc-13513, Santa Cruz Biotechnology, Inc.) (1:10), c-KIT/CD117 (#A4502 Dako) (1:200) and CLK-1 (#SAB1300108 Sigma-Aldrich) (1:100).

Corresponding TMA were acquired and digitalized using the Ariol SL-50 system coupled with fully automated microscope system Leica DM6000 B. Scores were generated using the TMASight assay, providing areas of high staining (Area_Color 1), medium staining (Area_Color 2) and low staining (Area_Color 3). We calculated the percentage of each staining per biopsy normalized to Area_Color 4, which represents the whole tissue, providing a computerized H-score calculated by formula: ((% of Area_Color1×3) + (% of Area_Color2×2) + (% of Area_Color 1×1))/100

### Immunoblots

Cells were washed 2× with PBS and harvested in cold RIPA Buffer (Sigma) containing 1% protease and phosphatase inhibitor cocktail (Halt EDTA-free; Thermo Scientific). Cell lysates were incubated at 4 °C for 15 min, sonicated for 15 min and clarified by centrifugation at 14,000 × *g* at 4 °C for 30 min. Protein concentration was estimated by a colorimetric assay (660 nm protein assay; Pierce) following the manufacture’s instruction. In total 20 μg of proteins per sample were loaded on 10% SDS–PAGE gel and transferred to nitrocellulose membranes for further processing. 5% BSA was used to block the membrane for 60 min at room temperature, followed by overnight incubation at 4 °C with the primary antibodies.

The following primary antibodies were used: phospho-AKT (Ser473) (D9E, #4060) (1:1000), phospho-p70S6K (Thr389) (#9205) (1:1000), p70S6K (#9292) (1:1000), phospho-p44/42 (ERK1/2) (Thr202/Tyr204) (#9101S) (1:1000), p44/42 (ERK1/2) (#9102) (1:1000), phospho-S6 Ribosomal Protein (Ser240/Ser244) (D68F8, #5364) (1:1000) and phosho-PNKP (Ser114/Thr118) (#3522S) (1:1000) (all from Cell Signaling Technology); CDK6 ready to use (clone 98D/H8, Monoclonal Antibodies Core Unit, CNIO), PKC Epsilon (EPR1482(2), #LS-B7689/36454) (1:1000), DAPK3/ZIPK (#LS-B557/43125) (1:1000) and phospho-CHK1 (Ser45) (#LS-C177888) (1:1000) from LifeSpan BioScience, c-KIT/CD117 (clone YR145, #04-214, Milipore) (1:1000), PNKP (#NBP1-87257, Novus biologicals) (1:1000), CLK-1 (#SAB1300108) (1:1000), Vinculin (#V9131) (1:5000) and β-Actin (clone AC-15, #A1978) (1:5000) from Sigma-Aldrich. Membranes were incubated with appropriate peroxidase-conjugate secondary antibodies (Sigma, St Louis, MO, USA). Bands were visualized by the enhanced chemiluminescence (ECL) method (Lumi-LightPlus detection kit; Roche). Uncropped versions of the blots can be found in Supplementary Figures 14–16.

### Proteomics sample preparation and runs

All tumor samples in study had >75% tumor content. We interrogated the phosphoproteome by enriching the phospho-fraction out of 250 micrograms of purified protein per sample by Ti4-IMAC chromatography coupled with mass-spectrometry. Given the large number of samples and the limited amount of material of the clinical specimens, we aimed to probe the phosphoproteome using a label-free single-shot strategy as described previously by de Graaf et al.^55^. Such approach allows highly reproducible measurements, both qualitatively and quantitatively, over a large dynamic range^55^.

Frozen tumor samples were homogenized using a Precellys 24 device (Bertin Technologies) in ice-cold lysis buffer containing 7 M urea (ThermoFisher Scientific), 2 M thiourea, 2% N-octyl glucoside (Santa Cruz Biotechnology, Inc.), 15 mM tris (2-carboxyethyl) phosphine (TCEP), 50 mM Hepes (pH 7.5), protease and phosphatase inhibitor cocktail (Halt EDTA-free; Thermo Scientific), and 0.1% Benzonase Nuclease (Novagen).

Tumor cell lines were washed 2 times with cold PBS 1× and harvested in ice-cold lysis buffer containing 7 M urea, 2 M thiourea, 2% N-octyl glucoside (Santa Cruz Biotechnology, Inc.), 15 mM tris (2-carboxyethyl) phosphine (TCEP), 50 mM Hepes (pH 7.5), protease and phosphatase inhibitor cocktail (Halt EDTA-free; Thermo Scientific), and 0.1% Benzonase Nuclease (Novagen).

Protein lysates from tumors and cells were sonicated for 15 min on ice and clarified by centrifugation at 14,100 × *g* at 4 °C for 15 min. Protein concentration was estimated by a colorimetric assay (660 nm protein assay; Pierce; Rockford, IL, USA) using bovine serum albumin as reference.

Protein samples were digested using the filter-aided sample preparation method^56^ Briefly, 250 μg of each tumor and cell line protein extract dissolved in lysis buffer was reduced with 10 mM DTT for 20 min at 56 °C and alkylated using 50 mM IAA solution for 20 min at 25 °C in the dark. The excess of reduction and alkylation reagents were washed with 8 M urea in 100 mM Tris (pH 8). The proteins were digested at 37 °C in wet chamber for 4 h using endoproteinase Lys-C at a 1:50 enzyme-to-protein ratio. After Lys-C digestion, urea was adjusted to 2 M with 50 mM triethyl ammonium bicarbonate. Trypsin (Promega) was added at a 1:50 (enzyme-to-protein ratio) and samples were subjected to a second digestion at 37 °C overnight in wet chamber. The digestion was quenched by acidification with formic acid (0.1% final concentration). Prior to phosphopeptide enrichment, digested samples were cleaned up with reversed phase-based solid-phase extraction (SPE). Phosphopeptide enrichment was performed using Ti4^+^-IMAC GELoader spin tips by centrifugation^57^. Briefly, Geloader tip microcolumns packed with Ti4 ± IMAC beads were created using a C8 plug at the constricted end. An aliquot of the Ti4 ± IMAC beads suspension was packed in the tip at the ratio 2:1 of beads:peptides. Max 250 µg of each digested tumor or cell line sample was loaded onto the column and centrifuged at 50 g for 30 min to allow the binding of phosphopeptides to the Ti4 + -IMAC beads (~3 µl/min). Bound phosphopeptides were washed with 80% acetonitrile (ACN) in 6% trifluoroacetic acid (TFA) by centrifugation at 170 g (~3 µl/min). Afterwards two additional washing step were performed, first one with 50% ACN/ 0.5%TFA in 200 mM NaCl and second one with 50% ACN/0.1% TFA. Both washing steps were performed by centrifugation at 170 g (~3 µl / min). Phosphopeptides were eluted from the column with 10% ammonia into 25% formic acid (FA) and centrifuged at 100 g (~1 µl/min). Finally, a second elution was carried out with 80% ACN/2%FA and centrifuged at 100 g (~1 µl/min). Phosphopeptides were acidified by adding 3 µl of 100% FA. The eluated was directly injected and analyzed by LC-MS/MS.

Peptides were subjected to reverse phase nano-LC-MS/MS analysis using a Proxeon EASY-nLC 1000 (Thermo Scientific) with an analytical column heater (40 °C) and an LTQ-Orbitrap Elite (Thermo Fisher Scientific). Peptides were first trapped (Reprosil C18, Dr Maisch, GmbH, Ammerbuch, Germany, 3 μm, 2 cm × 100 μm) at a maximum pressure of 800 bar with 100% solvent A (0.1% formic acid in water) before being separated on a 40 cm × 50 µm analytical column (Poroshell 120 EC-C18, 2.7 µm, Agilent, Santa Clara, CA). Peptides were chromatographically separated by a 90-min gradient from 7 to 30% solvent B (0.1% formic acid in ACN) at a flow rate of 100 nL/min. The total measurement time for each sample was 120 min. The eluent was sprayed via a distal coated fused silica emitter (360-μm outer diameter, 20-μm inner diameter, 10-μm tip inner diameter; constructed in-house) butt-connected to the analytical column. The electrospray voltage was set at 1.7 kV. The mass spectrometer was operated in a data-dependent mode to automatically switch between MS and MS/MS. Briefly, survey full-scan MS spectra were acquired in the Orbitrap analyzer, scanning from *m*/*z* 350 to *m*/*z* 1500 at a resolution of 60,000 at *m*/*z* 400 using an automatic gain control setting of 1e6 ions. Charge state screening was enabled, and precursors with either unknown or 1 + charge states were excluded. After the survey scan, the 20 most intense precursors were selected for subsequent decision-tree-based ion trap CID or ETD fragmentation^58^. The normalized collision energy for CID was set at 35%, and supplemental activation for ETD and dynamic exclusion were enabled (exclusion size list: 500; exclusion duration: 40 s).

### Proteomics data analysis and kinase prediction

The raw MS data were processed with MaxQuant software suite version 1.3.0.5^59^. The mass spectrometry proteomics data have been deposited to the ProteomeXchange Consortium via the PRIDE^60^ partner repository with the dataset identifier PXD008012The fragmentation spectra were searched against the *Homo sapiens* Uniprot database (downloaded on 23-12-2013), using Andromeda as the search engine. The precursor mass tolerances were set to 20 ppm for the first search and 4.5 for the main search. Also, 0.05 and 0.5 Da were used for FT and IT detectors. Carbamidomethylation of cysteine was considered as fixed modifications, whereas oxidation of methionine (M); phosphorylation on serine (S), threonine (T) and tyrosine (Y); and protein N-terminal acetylation were chosen as a variable modification, and up to two tryptic missed cleavages were allowed. The match between run function was enabled. A target-decoy database searching strategy was used to evaluate the false-discovery rates (FDRs) at the peptide and protein level.

The identification of kinase specific substrates was evaluated using linear sequence motifs analysis implemented in MaxQuant^59^. For the identification of phosphorylated motifs Maxquant used the PhosphoMotif Finder search tool at Human Protein Reference Database was used (http://www.hprd.org/PhosphoMotif_finder)^61^.

We applied the Consensus Clustering module using ConsensusClusterPlus package available in Bioconductor (http://www.bioconductor.org/) for class discovery and clustering validation. This method facilitates the discovery of biologically meaningful clusters assessing the stability for the discovered clusters by means of resampling techniques. Briefly, a clustering method chosen by the user is applied to each of the resampled data sets and, the consensus among the multiple runs, is assessed and summarized in a consensus matrix. This matrix is used as a visualization tool to estimate the cluster composition and number. For this study the Consensus Clustering analyses were run with hierarchical clustering algorithm, and Pearson correlation using 1000 resampling iterations.

Differentially expressed phosphopeptides were obtained by applying linear models with R limma package^62^ (Bioconductor project, http://www.bioconductor.org). To account for multiple hypotheses testing, the estimated significance level (*p* value) was adjusted using Benjamini & Hochberg False Discovery Rate (FDR) correction. Those phospho-sites with FDR < 0.15 were selected as differentially phosphorylated between classes under comparison. Representative Heat-Map was depictured using GENE-E software. (www.broadinstitute.org/cancer/software/GENE-E/)

A gene Set Enrichment Analysis (GSEA) was used to define set of kinase substrate motifs that shows statistically significant, concordant differences between relapsed and cured cases and aggressive and indolent triple-negative cell lines, herein termed Kinase-set enrichment analysis (KSEA). To this end, KSEA was applied using annotations for motifs extracted from Perseus software. Leading proteins were ranked based on their intensities. After Kolmogorov-Smirnoff testing, those kinase sets showing FDR < 0.05 were selected as significant^17^.

### GO pathways

Gene-ontology pathway analysis was performed with Enrichr: a comprehensive gene set enrichment analysis (http://amp.pharm.mssm.edu/Enrichr/#)^63,64^.

### Next generation sequencing

Massive parallel sequencing was performed using a custom panel of 100 genes (Entire Transcribed Region) with a size of 844Kbp. DNA was extracted from 113 FFPE samples using High Pure FFPET DNA isolation kit (Roche Diagnostics). Quality of DNA samples was measured by OD 260/280 ratio ranging from 1.8 to 2.0, and also by 2100 Bioanalyzer and DNA 1000 Assay (Agilent Technologies).

Processing of samples for sequencing was done using Sure Select XT Target Enrichment System for Illumina Paired-end Multiplexed Sequencing Library (Agilent Technologies). Genomic DNA was sheared to a fragment size between 120 and 150 bp using Covaris E-series. In total 400 ng of shared DNA were repaired the ends, adenylated the 3′ end of the DNA fragments and ligated the pared–end adapter according to manufacturer’s protocol. DNA were purified using AMPureXP beads (Beckman Coulter Genomics).

The captured libraries were amplified with indexing primers containing 8 bp indexes (Supplementary Data 4) and quality and quantity analysis were done using 2100 Bioanalyzer and DNA 1000 Assay (Agilent Technologies), selecting the DNA with a fragment size peak of ~225–275 bp and the concentration was determined by integration under the peak.

gDNA libraries were hybridized with a target-specific Capture Library in 75 samples out of 113 due to quality and quantity of DNA after DNA extraction and sequential purification steps. The targeted molecules were captured with streptavidin beads (Dynabeads MyOne Streptavidin T1, Life Technologies). Quality analysis of indexed libraries were done using the 2100 Bioanalyzer and High Sensitivity DNA Assay (Agilent Technologies) selecting the DNA with a fragment size peak of approximately 250 to 350 bp, concentration was measure by Qubit Assay (Qubit BR dsDNA Assay Kit, Life technologies). Libraries were combined into pools of 10 nM. Samples were sequenced by a HiSeq 2500 powerful high-throughput sequencing system. We used a flowcell rapid run in a format 100 bp paired-end reads (2 × 100PE). 69 samples were correctly sequenced with an average of coverage of 278×.

Fastq Data generated by the sequencing was aligned to the hg19 human reference sequence. Data were analyzed using RUbioSeq^65^, additionally MuTect^66^ was executed in order to identify variants with low frequency undetectable with RUbioSeq pipeline. Variants with sufficient coverage and good quality metrics were annotated using the Ensembl Variant Effect Predictor (VEP)^67^ with Ensembl v83. We also added information about pathogenicity from ClinVar database^68^ and COSMIC^69^ frequencies and pathogenic prediction for each variant. Using the annotations and visual inspection we removed possible artefacts that survived the variant calling filters. Polymorphisms were also discarded using the population frequencies of the 1000 Genomes Project and ExAC provided by VEP. From the remaining set of variants we selected the most relevant using the pathogenic impact and the clinical evidences (Variants described in ClinVar and COSMIC with pathogenic prediction and also variants with a relevant high impact in SIFT, PolyPhen or Condel).

### Statistics

In order to study the impact of the enriched kinases, their H-scores (continuous variables) were categorized to allow Kaplan-Meier analysis^70^. The kinase scores were categorized as follows: patients with a kinase staining above the 75th percentile were encoded as “1”, whereas the remainder were encoded as “0” (both for the Kaplan–Meier tests and the Cox models). The kinases with positive association with relapse were grouped into a single variant (K-high); patients with a staining in one or more (positively associated) kinases above the 75th percentile were encoded as K-high = 1; in order to be encoded as K-high = 0, patients had to have a staining below the 75th percentile in all six kinases. Both the prognostic value of each candidate kinase and that of K-high was calculated in two ways: with the Kaplan Meier method and the Log-Rank test (univariate), and with the Cox’s proportionate hazards model (multivariate model, adjusting the hazard ratio attributable to each kinase by the T and N status, age and grade), implementing a multiple comparisons correction (Bonferroni) to the cut-off point of *P* < 0.05. In addition, prognosis accuracy of K-high was compared with other variables (K-test) resulting from the combination of 2, 3, 4 or 5 kinases with staining above the 75th percentile. Nine different variables (K-test_1_ to K-test_9_) were generated and Kaplan–Meier curves, Log-Rank tests and multivariate Cox’x analysis were run for each test variable corrected by multiple comparisons effect (Bonferroni). Kinase co-linearity was investigated with the Pearson´s coefficient in a pairwise manner.

Tumor burden comparisons and survival benefit between treatment groups for the in vivo experiments were performed with a *t*-test (average burden obtained from at least 10 tumors per time point and condition) and the Kaplan–Meier method (used to compare the time-to-sacrifice between treatment groups). The minimum number of animals per group was calculated in order to detect a minimum of 30% variation in the time to sacrifice (institutional guidelines set mandatory sacrifice when tumors grow >1000 mm^3^), based on the medium time to sacrifice and standard deviation of the vehicle-treated animals and setting alpha and beta errors in 5% and 20%, respectively. All tests were performed with the SPSS Statistics V.19.0 software.

## Electronic supplementary material

Supplementary Information

Description of Additional Supplementary Files

Supplementary Data 1

Supplementary Data 2

Supplementary Data 3

Supplementary Data 4

Supplementary Data 5

## Data Availability

The proteomics data have been deposited to the ProteomeXchange Consortium via the PRIDE partner repository with the dataset identifier PXD008012. Genomics data have been deposited in the database of the NCBI Sequence Read Archive under the accession code SRP152502. All other data supporting the findings of this study are available from the corresponding author on reasonable request.
