# Supplementary material for: In vivo phosphoproteomics reveals kinase activity profiles that predict treatment outcome in triple-negative breast cancer
Source: Nat Commun. 2018 Aug 29;9:3501. doi: 10.1038/s41467-018-05742-z (PMC6115463; doi:10.1038/s41467-018-05742-z)
Supplement: Supplementary file 1 — Supplementary Information [file 41467_2018_5742_MOESM1_ESM.pdf]

In vivo phosphoproteomics reveals kinase activity profiles that predict treatment outcome in triple-negative breast cancer

Zagorac et al.

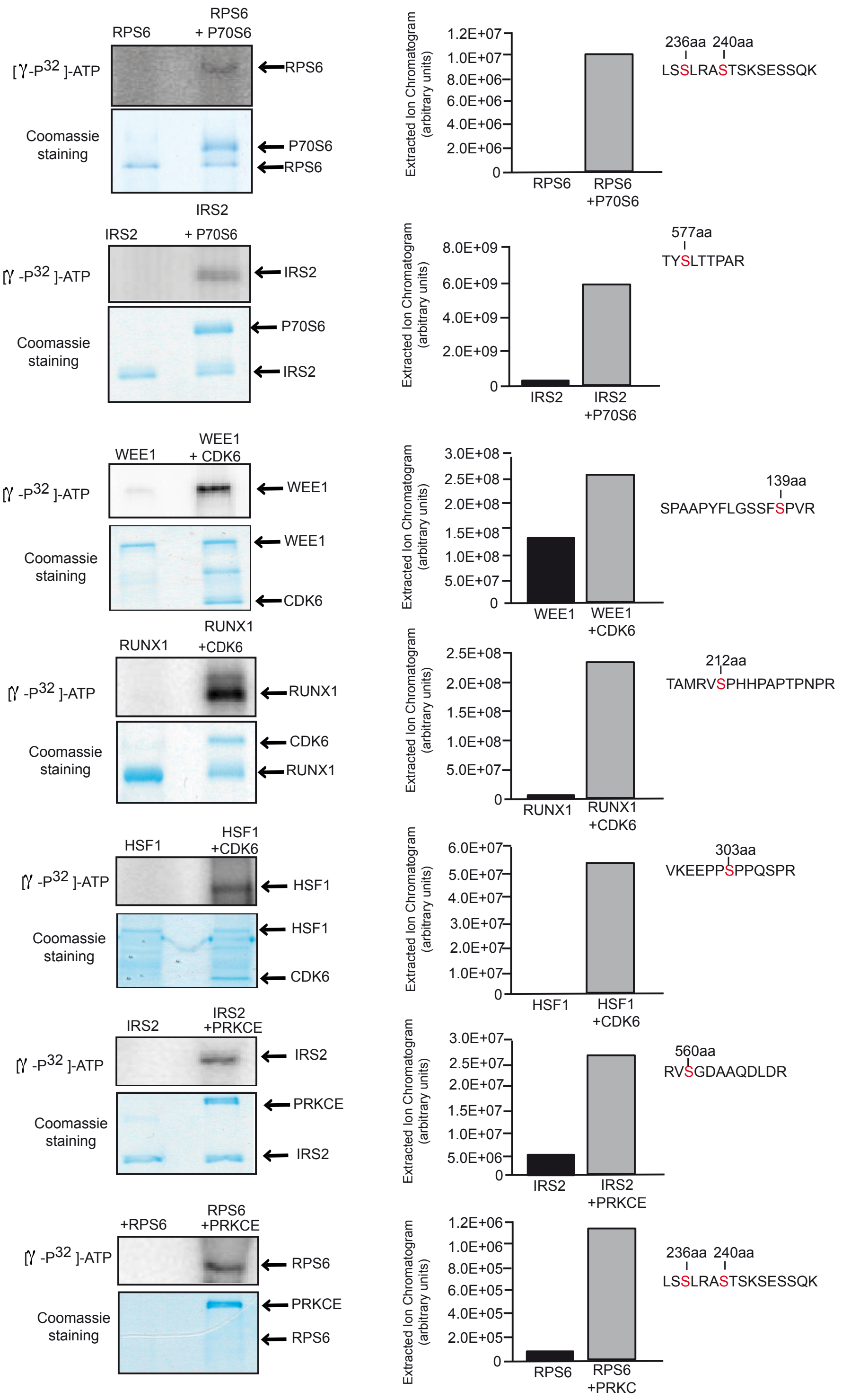

Supplementary Figure 1. Substrates used for building KSEAS charts are *bona fide* substrates of their predicted upstream kinases. Three kinases of the final signature ("K-high") were extracted from KSEAS predictions (namely, PRKCE, P70S6K and CDK6). We tested several random substrates predicted to be phosphorylated by such kinases according to the KSEAS algorithm. In the left hand side, for each substrate, an upper panel showing the P^{32}-ATP autoradiography of the substrate (left) and the kinase plus the substrate (right) after performing the kinase assay is shown Also for each substrate, in the lower panel, a picture of the Coomassie staining of the gels containing the substrate alone (left) and the kinase plus the substrate (right) run after the kinase assay without radioactive P is depicted. In the right hand side, for each substrate, spectral counts of the highlighted residues of the depicted peptides that map towawrds each of the substrates of interest containing a phosphate group are plotted. The spectral counts of the charts left hand side column correspond to the mass spectromerty runs of the protein-substrate isolated from the band of the Coomassie staining from the kinase-free reactions, whereas the right hand side columns of the chart correspond to the bands obtained from the reactions with the kinase.

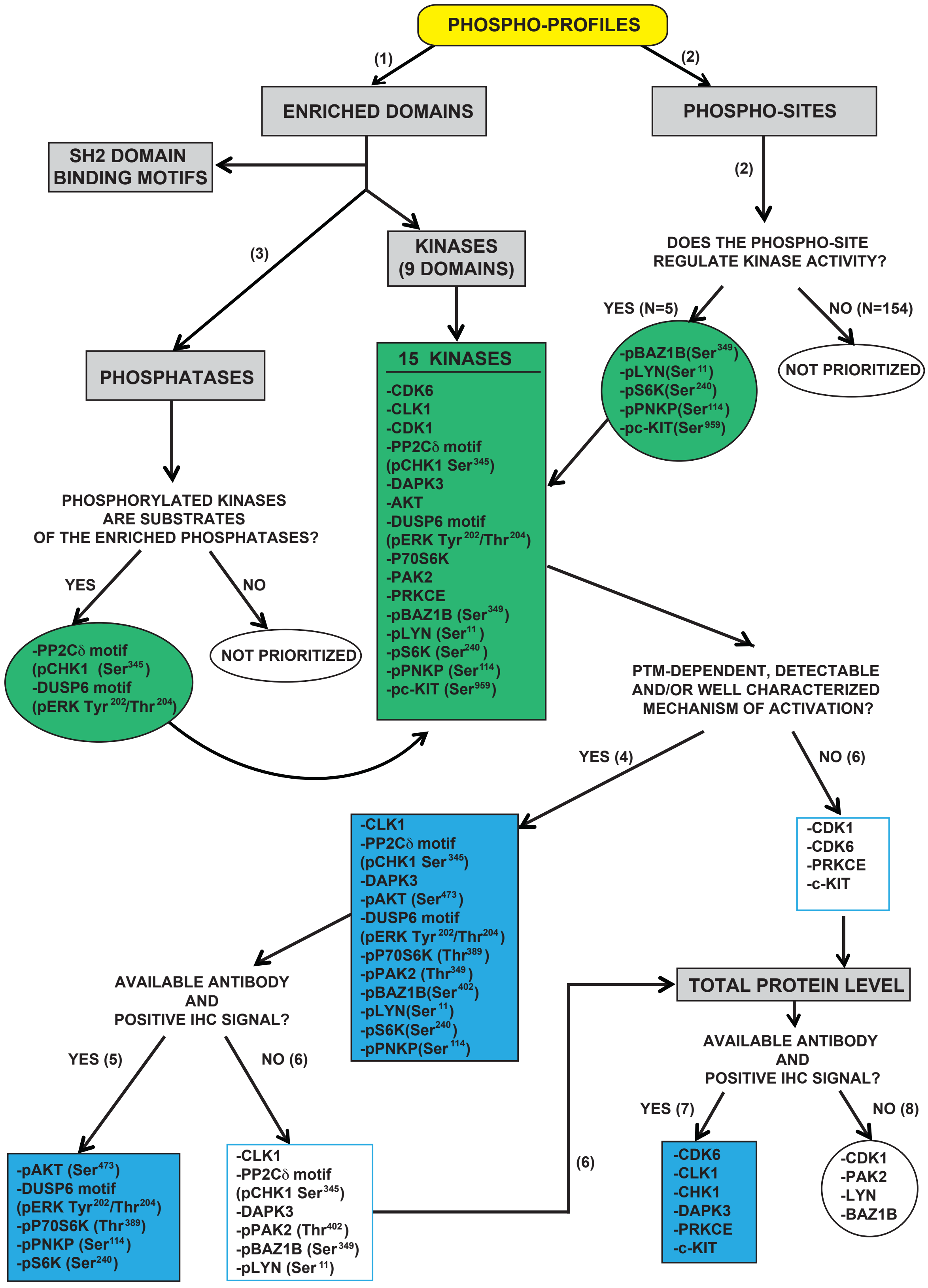

Supplementary Figure 2. Algorithm for the selection of antibodies against enriched/activated kinases. These antibodies were subsequently used on the 113 samples from the validation set. To define an "activated kinase" and detect it in a formalin-fixed, paraffin-embedded (FFPE) sample are complicated tasks. The *in silico* algorithm maps the phosphosites of the relapsed cases/aggressive cell lines to their driver

kinases, indicating that if a given kinase is enriched in a profile, it theoretically shows high catalytic activity as compared with its activity in non-relapsed cases/indolent cells lines. The activity of a suspect kinase can only be indirectly assessed in FFPE samples, and requires a case-by-case set-up. An easy-to-set-up kinase detection case would be AKT, where the detection of phosphorylation at pAKT(Ser^{473}), a post-translational modification (PTM) associated with a several-fold increase in catalytic activity[1], in a given sample would imply increased AKT activity as compared with a different sample with non-phosphorylated AKT. Other kinases are difficult to assess in FFPE samples including those that; do not undergo functional modulation based on PTM (PIM[2]), or a complex, transient, and sometimes incompletely understood chain of events leading to activation (c-Kit[3] or PRKCE, where different PTMs can induce opposing modulation[4]) or can be activated by changes undetectable by immunohistochemistry (activating mutation in c-Kit[3] or calcium signalling in PRKCE[5]). In light of these difficulties, a more conservative approach, such as measuring total kinase levels, is more appealing. The depicted algorithm shows the list of steps that we followed to identify the antibodies that should detect, as accurately as possible, the activated kinases being studied here.

(1) Enriched kinase domains predicted from the phosphoprofiles were prioritised over phosphosites, since they provide a more functional portrait of the effector layer. In addition, the present study aimed to elucidate classifiers that were simultaneously therapeutically targetable enzymes; thus, non-kinase hits were de-prioritised. However, **(2)** some of the phosphosites from the list of 159 upregulated sites (Table S2) actually corresponded to kinases, and kinase function is often regulated by phosphorylation. Thus, 5 kinases were added to the testing workflow.

Some of the enriched domains corresponded to phosphatases **(3)** instead of kinases (DUSP6 and PP2Cδ), which reflects the abundance of a phosphorylated substrate that can be cleaved by the identified phosphatase; simultaneously such a phosphosite/substrate could be difficult to detect in high abundance due to the activity of phosphatases. If one of those substrates was a phosphorylated site in a kinase, its presence was verified in the phospho-data matrix and added to the testing workflow. A total of 15 kinases (9 from the KSEAS, 2 from the phosphatases and 5 from the Table S2 phosphostes; however, S6K was repeated in the KSEAS and in Table S2) were therefore selected for the validation experiment.

If a given kinase had a well-described, dominant, and theoretically detectable mechanism of activation (phosphorylation at S473 in the case of AKT[1]) **(4)**, antibodies against that target were tested first in pelleted cell lines, (FFPE-processed in the same way as human tumour samples) with a known status of the targets, by western blotting. Antibodies useful for IHC signals were then tested in control human samples, and if a positive signal was observed, the antibody probes were considered adequate for the investigation of the validation tissue microarray (TMA) set. Five hits were selected through this workflow **(5)**. Antibodies against the nonphosphorylated forms of the kinases for those that failed detection or did not have an antibody available suitable for IHC in human tissue were subsequently tried **(6)**. Three more types of kinase were probed with antibodies against the total protein as follows **(6)**: 1: kinases for which no further regulation of transcriptional levels accounted for activation (CDK1/6); 2: kinases for which a non-IHC-detectable PTM accounted for the activation state (calcium signalling, lipidic modifications, AMP, allosteric modifications, mutations; PRKCE[5, 6] or c-Kit[3, 7, 8]); 3: kinases for which activation is driven by complex and transient mechanisms with opposing actions (PRKCE[4] or c-Kit[9, 10]). The same antibody set-up workflow (western blotting, cell-IHC, and finally tissue IHC) was applied, which led to a further 6 probes suitable for detection **(7)**, and 4 probes were finally discarded **(8)**.

The following reasons may account for the detection of an activated kinase in the list of phosphosites (Table S2) having not been predicted from consensus domain analysis of the phosphoprofiles (Figure 2), or vice-versa (as opposed, for instance, to S6K, which was predicted from both the consensus domain analysis of the phosphoprofiles and was detected the list of phosphosites, where it appeared to be in a phosphorylated form [Ser240]): 1) the definition of consensus domains is knowledge-based; despite the fact that other potential substrates can be postulated from the sequences of the already-proven kinase substrates, the accuracy and specificity of this process will be refined with increasing knowledge[11, 12] (implying, for instance, that substrates of PNKP are present in the phosphoprofiles of aggressive tumours, but since those substrates are still uncharacterised or unknown, the activated PNKP is not predicted from the consensus domain analysis; in contrast, phosphorylated PNKP was found in the phosphosites in Table S2); 2) the temporal regulation by phosphatases and other feedback signalling loops is not integrated into the analysis, meaning that a kinase can be activated, but its substrates may or may not be detected due to the dynamic nature of signalling events; and 3) the depth of coverage of the proteome may be insufficient. Although more than 15,000 phosphosites were identified in the present study, the number of residues that can be phosphorylated in the human proteome can be at least 1 order of magnitude larger. Therefore, kinase mapping could be further refined in the future.

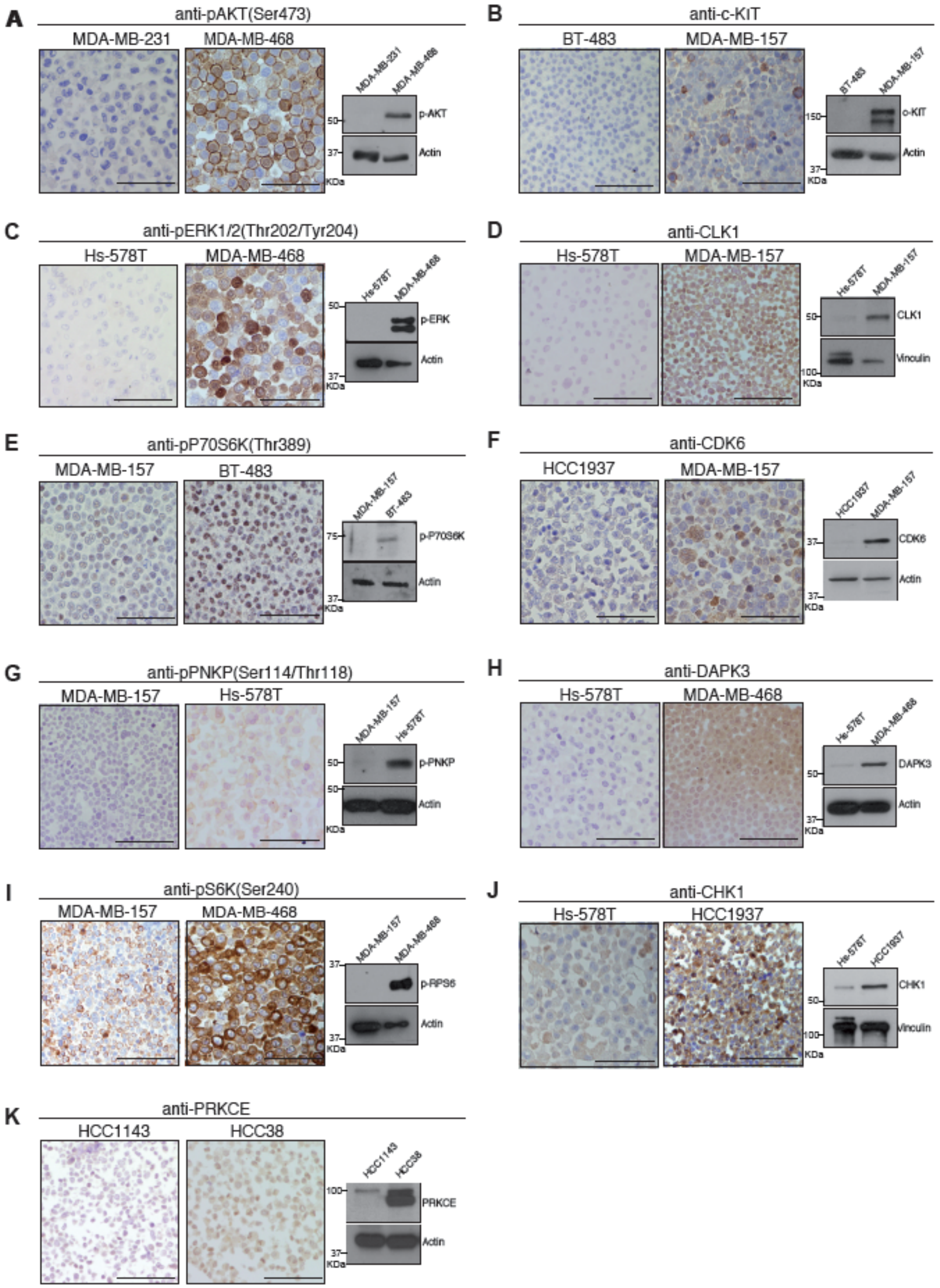

Supplementary Figure 3. Control stainings. TNBC cell lines were pelleted, fixed with formalin, and embedded in paraffin blocks. Pellet sections were stained following the same protocols as those applied to the TMAs. Panels **(A)** to **(K)** show representative IHC-stained sections of cell line pellet pairs positive or negative for different proteins (i.e., such as c-KIT in B for BT-483 /MDA-MB-157), or with substantially different protein levels (i.e., such as CHK1 in J for Hs578T/HCC1937) used to discriminate the sensitivity and specificity of the 11 antibodies used in the validation set. Scale bars 50μm.

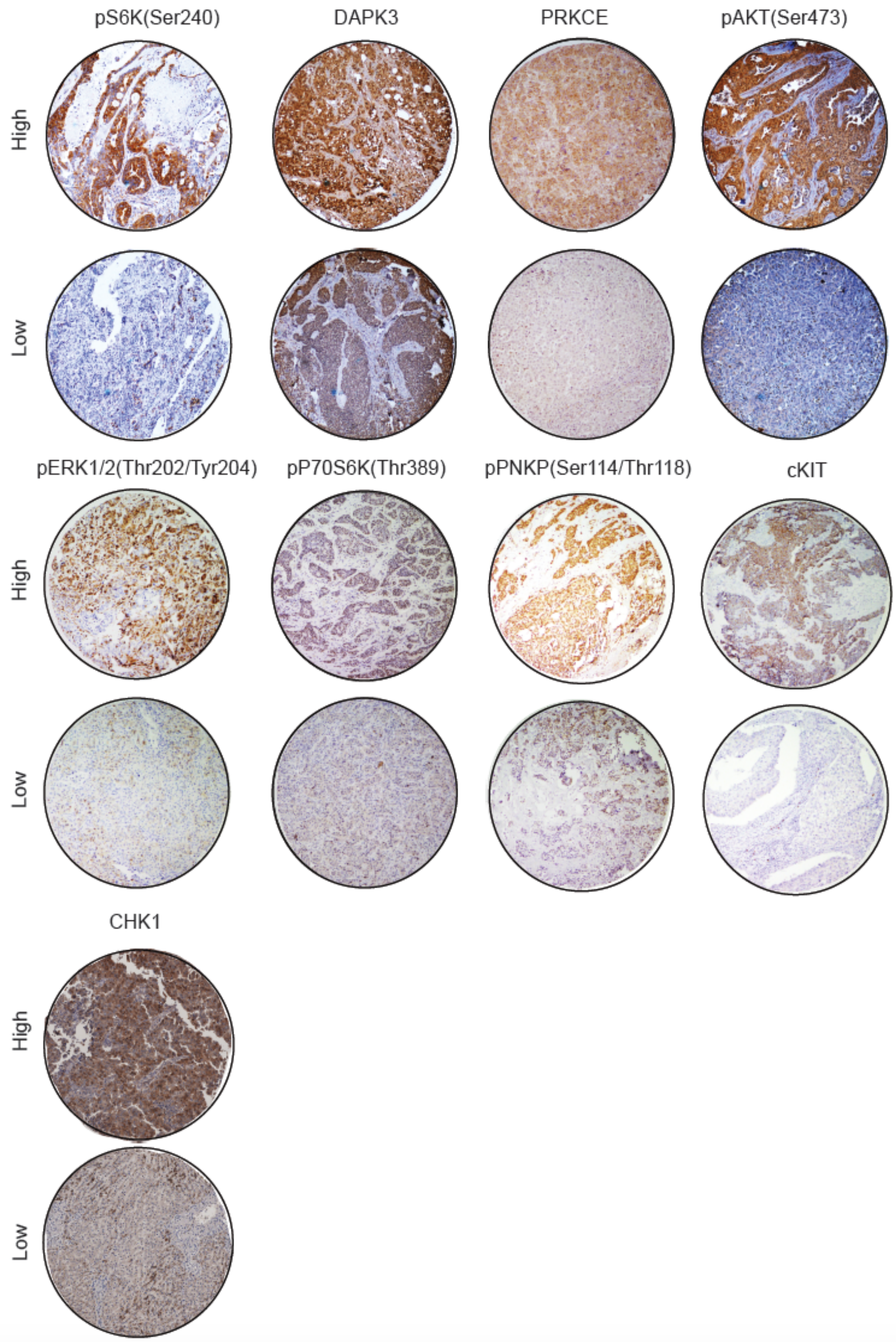

Supplementary Figure 4. Representative tumour cores stained in the validation set. For each kinase, a TMA-core with staining yielding an H-score above the upper quartile and a core with an H-score below the lower quartile are shown. The H-score values defining the upper, medium, and lower quartiles for all the probes are shown in Supplementary Table 3.

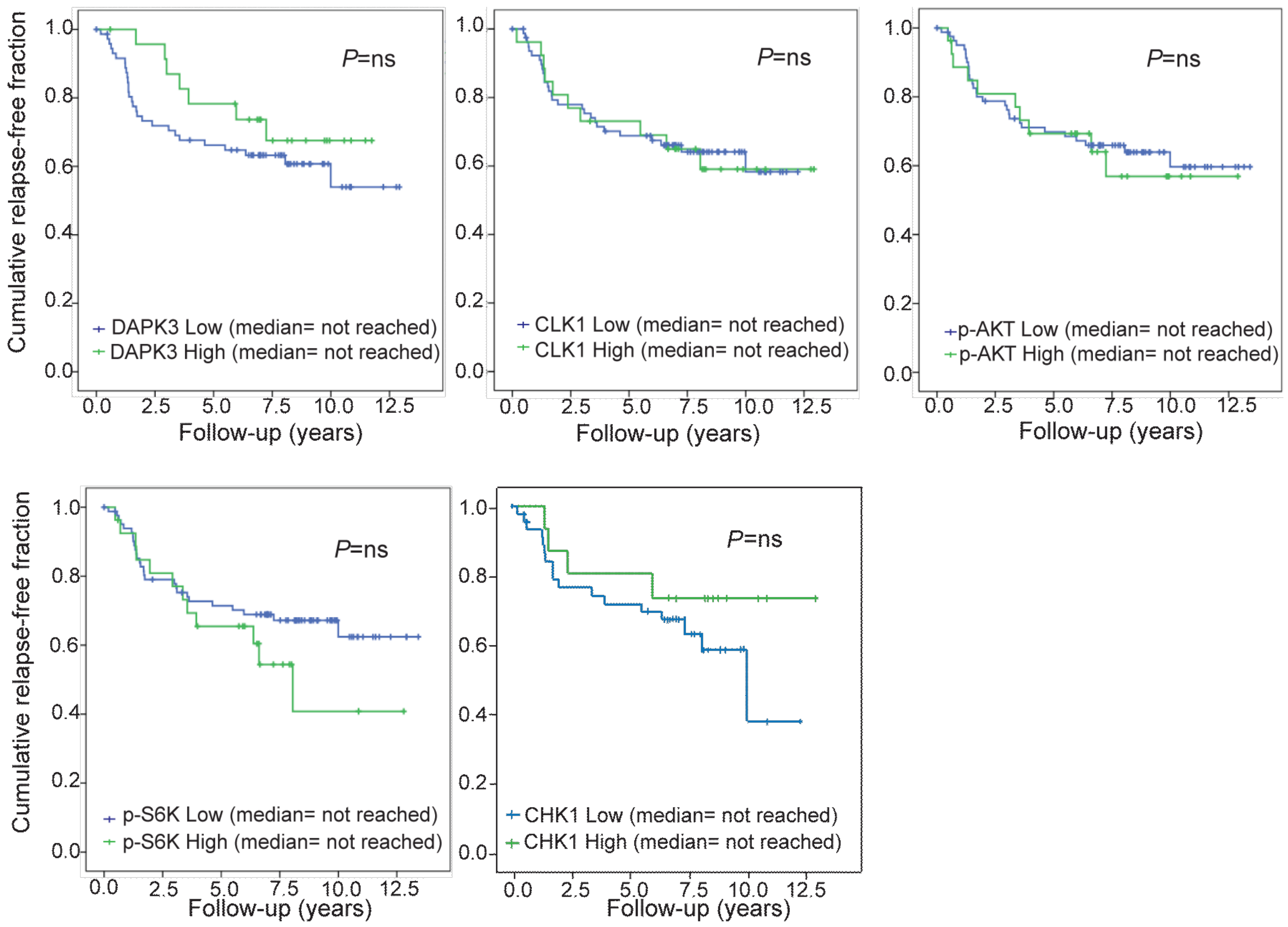

Supplementary Figure 5. Kinases without significant prognostic association in the validation set. Kaplan-Meier curves for DAPK, CLK1, p-AKT, p-S6K, and CHK1. Neither the log-rank tests nor the adjusted hazard ratios in the Cox analysis were statistically significant in any of the 5 cases.

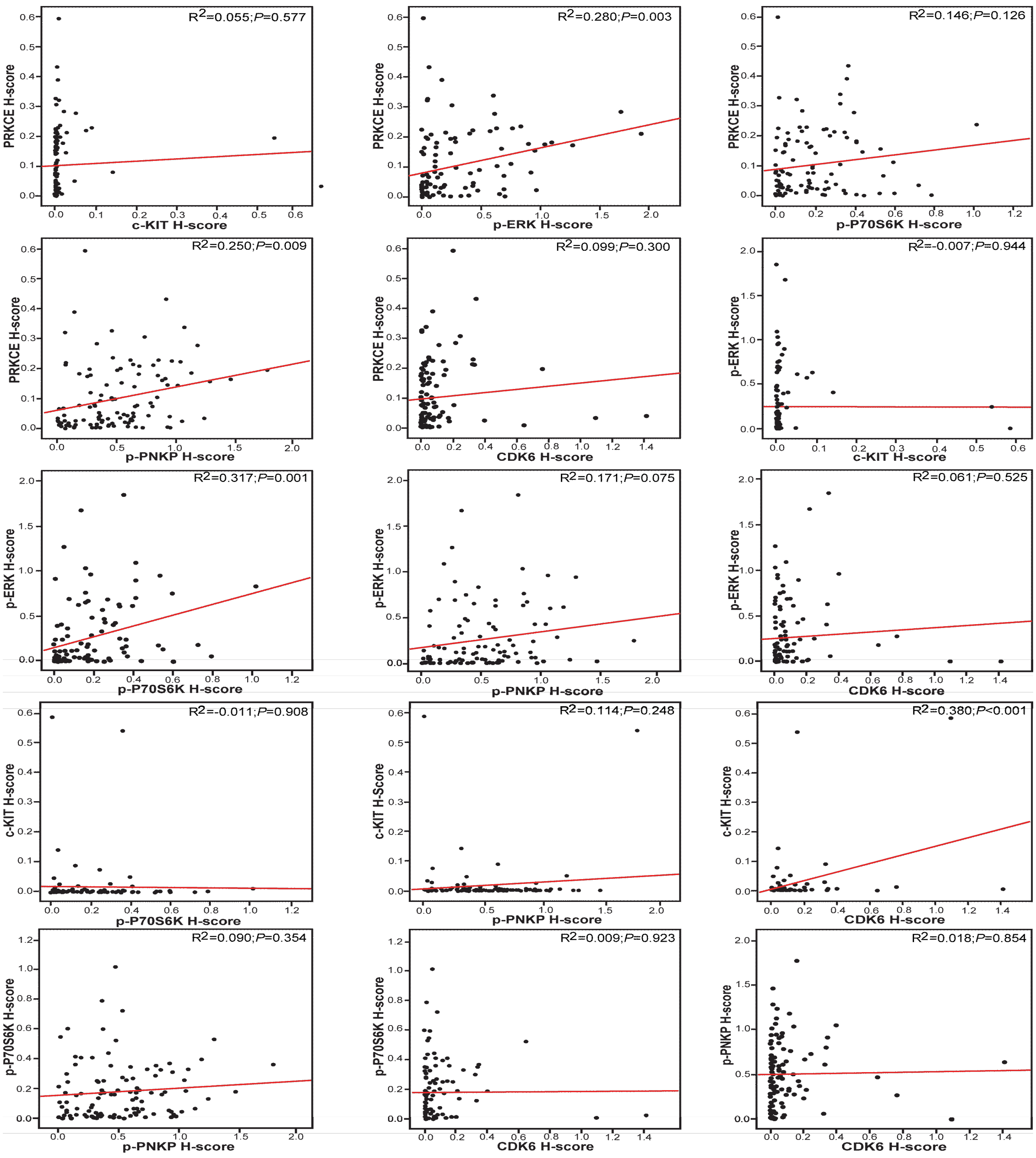

Supplementary Figure 6. Lack of statistically significant colinearity between the prognostic 6 kinases. The H-scores of the 6 kinases found in each of the patients within the validation set are plotted in a pairwise manner. Each chart shows the correlation between the H-score of two kinases. Chart dots (one dot per patient) represent the individual H-score for each of the two kinases, placed with respect to the X and Y axes according to each of the two H-scores. An R^2-Pearson coefficient was calculated for the correlation between the H-score of each kinase versus any other. Among the 15 possible correlations between the 6 kinases, 11 were not statistically significant. The remaining 4, although significant, had very low R^2 coefficients. We could not find available published evidence that established an R^2 cut-off that could differentiate "linear-related" kinases in the same pathways from those that are functionally unrelated. However, correlations between 0.3 and 0.5 are considered "weak"; between 0.5 and 0.7 are considered "moderate", and greater than 0.7 are considered “strong”. Two of the pairs with statistical significance (PRKCE/p-ERK and PRKCE/p-PNKP) had an R^2 less than 0.3, and the other 2 pairs (p-ERK/p-P70S6K and c-Kit/CDK6) had an R^2 less than 0.4. The correlation between c-Kit and CDK6 appeared strongly affected by a single outlier value, and no published reports have shown reasonable evidence linking the two pathways. Cross-talk has been reported for the Pi3K-AKT-MTOR and MAP-kinase pathways; however, although they show some common downstream pathways, their functions are mostly independent[13].

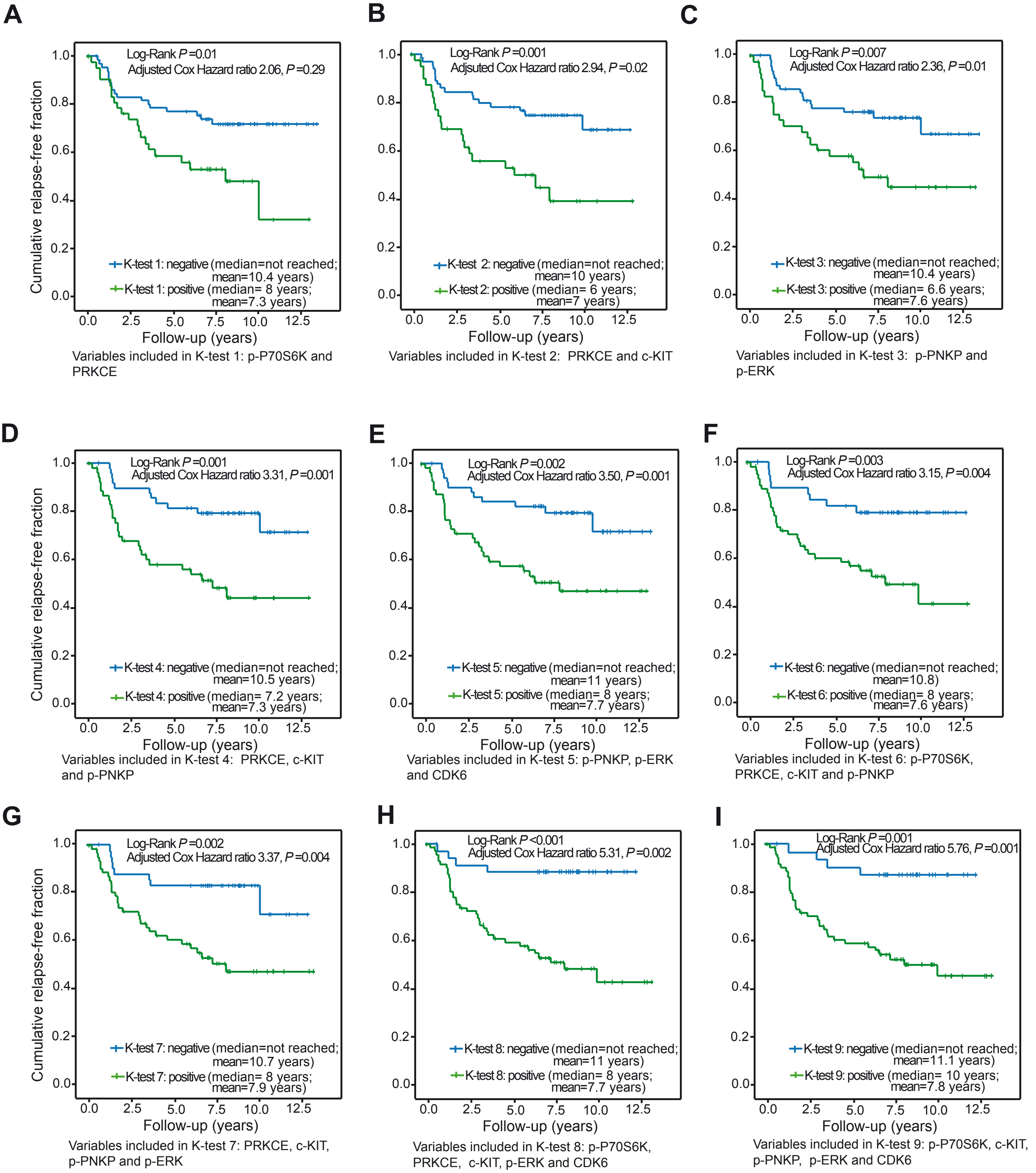

Supplementary Figure 7. Prognostic impact of test variables. Nine different test variables were generated (K-test$_1$ to K-test$_9$). Variables were built as follows: if a given patient had H-scores for all the kinases that were considered in that variable above the 75th percentile, K-test$_n$ was encoded as "1". In any other case, K-test$_n$ was encoded as "0". For instance, K-test$_1$ was built on the basis of the H-score of pP70S6K and PRKCE; thus, patients with upper-quartile staining of both kinases were encoded as K-test$_1$=1 ("positive"), whereas the remaining patients were encoded as K-test$_1$=0 ("negative").

Panels **(A)** to **(I)** show, respectively, the Kaplan-Meier curves, log-rank *p*-values, and Cox´s hazard ratios (adjusted for T, N, age and G) corresponding to K-test$_1$ to K-test$_9$. Kinases included in building each K-test$_n$ are detailed below each curve. It can be observed that the clinical course separation, between the patients positive versus negative for test variables composed of 2 (A, B, and C), 3 (D and E), 4 (F and G), or 5 (H and I) kinases, progressively increases in conjunction with an improvement in *p*-value; however, none reaches the accuracy of K-high (Figure 4). All *p*-values are corrected for multiple comparisons (Bonferroni correction).

A

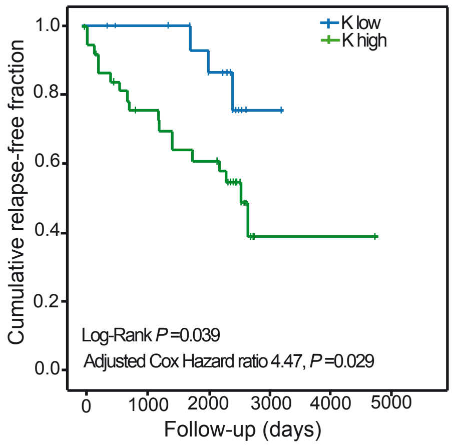

B

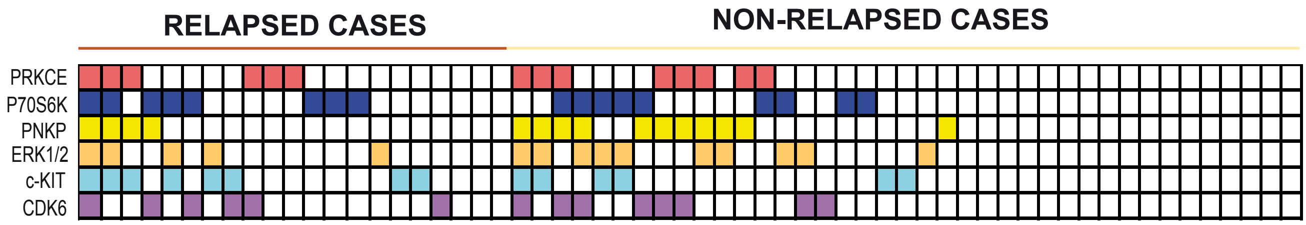

Supplementary Figure 8. The role of K-high in a second independent validation set (Val-2). An independent set consisting of TNBC patients (n = 61) who received current standards of care in the adjuvant setting (anthracyclines, cyclophosphamide, and taxanes) was gathered. Supplementary table 1 shows the clinical and pathological characteristics of these patients. In this series, only the 6 kinases from K-high were analysed, to serve as a validation set for the mass-spectrometry-to-immunohistochemistry translation step (training set to Val-1), while increasing the total number of patients for validation. **(A)** Kaplan-Meier curve for the relapse of K-high versus non-K-high patients. **(B)** Grid showing the kinase activation pattern (upper quartile versus lower) in relapsed and non-relapsed patients from Val-2. The patterns are summarised in Supplementary data 3.

A

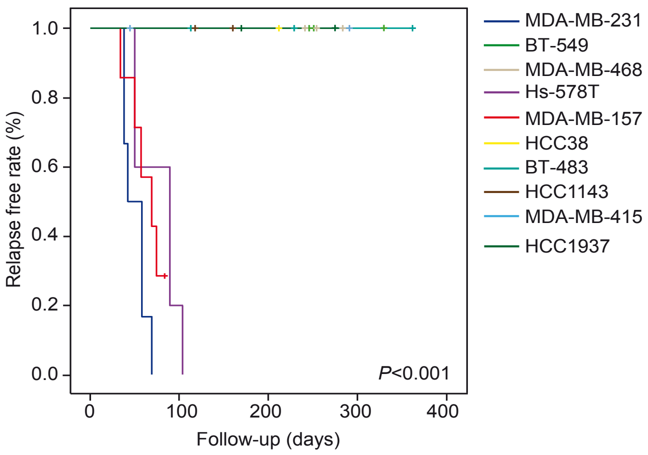

TNBC CELL LINE	MEDIAN TIME TO DEATH (days)
MDA-MB-231	50
MDA-MB-157	69
Hs-578T	90
MDA-MB-468	not reached at 254
HCC1937	not reached at 275
HCC1143	not reached at 160
HCC38	not reached at 362
BT-549	not reached at 251
BT-483	not reached at 229
MDA-MB-415	not reached at 291

B

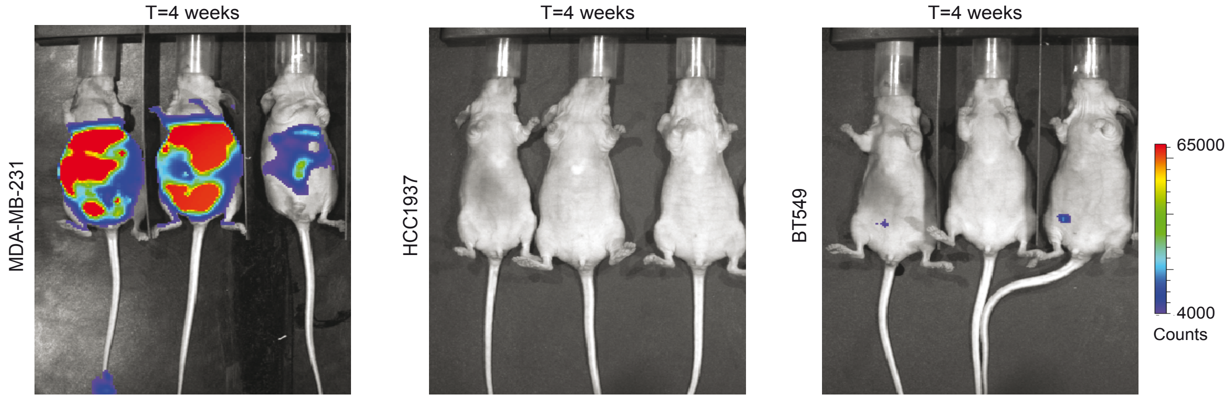

Supplementary Figure 9. Characterisation of human TNBC cell lines. (A) Left: Kaplan-Meier survival curves of mice (n = 60) intraperitoneally injected with different triple-negative breast cancer (TNBC) cell lines. The intraperitoneal injection of tumour cell lines is a stringent model of metastasis compared with tail-vein injection; since tail vein injection omits the step during which tumour cells leave the primary site and enter the bloodstream. Right: Median time to sacrifice of the groups of mice injected with each of the cell lines (log-rank test, $p < 0.001$). TNBC cell lines were classified as “aggressive” (MDA-MB-231, MDA-MB-157, and Hs-T578) or “indolent” (MDA-MB-468, HCC1937, HCC1143, HCC38, BT549, BT483, and MDA-MB-415), according to their ability to kill as a result of loco-regional growth and the generation of metastases in less than 100 days or not (in more than 400 days), respectively. **(B)** Representative images of tumour burden and dissemination in animals 4 weeks after injection of one of the aggressive TNBC cell lines (MDA-MB-231, left) and two of the indolent cell lines (HCC1937 and BT549) are shown.

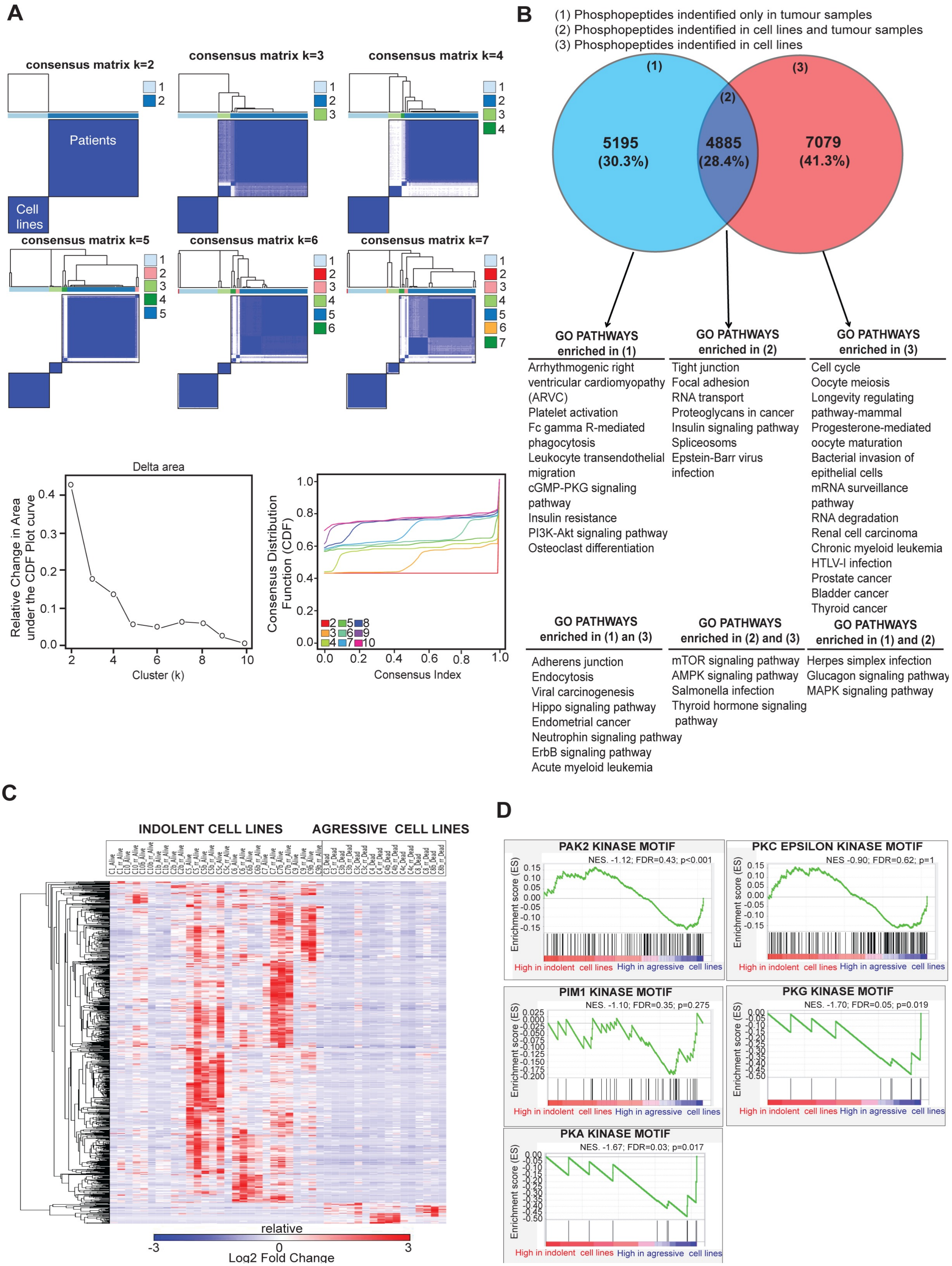
A
consensus matrix k=2
consensus matrix k=3
consensus matrix k=4
Patients
Cell lines
consensus matrix k=5
consensus matrix k=6
consensus matrix k=7
Delta area
Relative Change in Area under the CDF Plot curve
Cluster (k)
Consensus Distribution Function (CDF)
Consensus Index
B
(1) Phosphopeptides indentified only in tumour samples
(2) Phosphopeptides indentified in cell lines and tumour samples
(3) Phosphopeptides indentified in cell lines
5195 (30.3%)
4885 (28.4%)
7079 (41.3%)
GO PATHWAYS enriched in (1)
Arrhythmogenic right ventricular cardiomyopathy (ARVC)
Platelet activation
Fc gamma R-mediated phagocytosis
Leukocyte transendothelial migration
cGMP-PKG signaling pathway
Insulin resistance
PI3K-Akt signaling pathway
Osteoclast differentiation
GO PATHWAYS enriched in (2)
Tight junction
Focal adhesion
RNA transport
Proteoglycans in cancer
Insulin signaling pathway
Spliceosoms
Epstein-Barr virus infection
GO PATHWAYS enriched in (3)
Cell cycle
Oocyte meiosis
Longevity regulating pathway-mammal
Progesterone-mediated oocyte maturation
Bacterial invasion of epithelial cells
mRNA surveillance pathway
RNA degradation
Renal cell carcinoma
Chronic myeloid leukemia
HTLV-I infection
Prostate cancer
Bladder cancer
Thyroid cancer
GO PATHWAYS enriched in (1) an (3)
Adherens junction
Endocytosis
Viral carcinogenesis
Hippo signaling pathway
Endometrial cancer
Neutrophin signaling pathway
ErbB signaling pathway
Acute myeloid leukemia
GO PATHWAYS enriched in (2) and (3)
mTOR signaling pathway
AMPK signaling pathway
Salmonella infection
Thyroid hormone signaling pathway
GO PATHWAYS enriched in (1) and (2)
Herpes simplex infection
Glucagon signaling pathway
MAPK signaling pathway
C
INDOLENT CELL LINES
AGRESSIVE CELL LINES
relative
Log2 Fold Change
D
PAK2 KINASE MOTIF
NES. -1.12; FDR=0.43; p<0.001
PKC EPSILON KINASE MOTIF
NES -0.90; FDR=0.62; p=1
PIM1 KINASE MOTIF
NES. -1.10; FDR=0.35; p=0.275
PKG KINASE MOTIF
NES. -1.70; FDR=0.05; p=0.019
PKA KINASE MOTIF
NES. -1.67; FDR=0.03; p=0.017
Enrichment score (ES)
High in indolent cell lines
High in agressive cell lines

Supplementary Figure 10. TNBC cell line profiling and similarities to the training set. TNBC cell lines were phospho-purified and run alongside the human tumours in the training set. More than 11900 phosphopeptides, which mapped to at least 3100 proteins, were identified (Supplementary Table 2). **(A)** A consensus clustering algorithm was applied to estimate the number of clusters present in the phosphomatrix. The consensus matrix obtained from k = 2 to k = 7 (upper panel), the delta area plot, and the consensus Cumulative Distribution Function (CDF) plot (bottom panel) showed that the optimum number of clusters was 5. More specifically, we found two main groups containing 75 and 45 successfully classified tumours and cell lines, respectively. The additional 3 clusters were considered outliers. Each mass spectrometry run was considered a "sample" for this algorithm. This clustering suggests a separation between the nature of the phosphopeptides identified in the cell lines and those identified in the tumour samples. **(B)** Venn diagram of the phosphopeptides and Gene Ontology-related (GO) pathways enriched in the cell lines, tumours, or both. Thus, despite the consensus clustering data, the gene-ontology (GO) pathways enriched in the proteins to which the peptides mapped showed important overlap even for the nonredundant PSMs, with only a few GO pathways unique to either the cell lines or the tumour samples. GO analysis is inherently biased in the present study, since it originated from phosphorylated (which represent 1-2% of the proteome) instead of native peptides; it is not possible to ascertain whether the phosphorylated species isolated from the TiO2-IMAC chromatographs adequately represent all the proteins that were expressed or whether they were biased due to the activities of various kinases. The reason these data are presented is to show that beyond the 28.4% peptides that were identified in both cell lines and tumours, GO-pathway overlap was also found with the peptides that were isolated only in cell lines or in tumours, indicating a further degree of overlap. **(C)** Heatmap of the cell lines (stratified by degree of aggressiveness in nude mice). There were 874 peptides with significant differences in phosphorylation levels: 53 upregulated and 821 downregulated in the aggressive cell lines. These peptides can be found in the raw data matrix uploaded to the PRIDE database (accession number: PXD008012). **(D)** The KSEAS of the aggressive cell lines; enrichment for two (PAK2 and PRKCE) is shared with the human TNBC tumours, indicating a degree of overlap between the cell lines and tumours beyond the GO pathways.

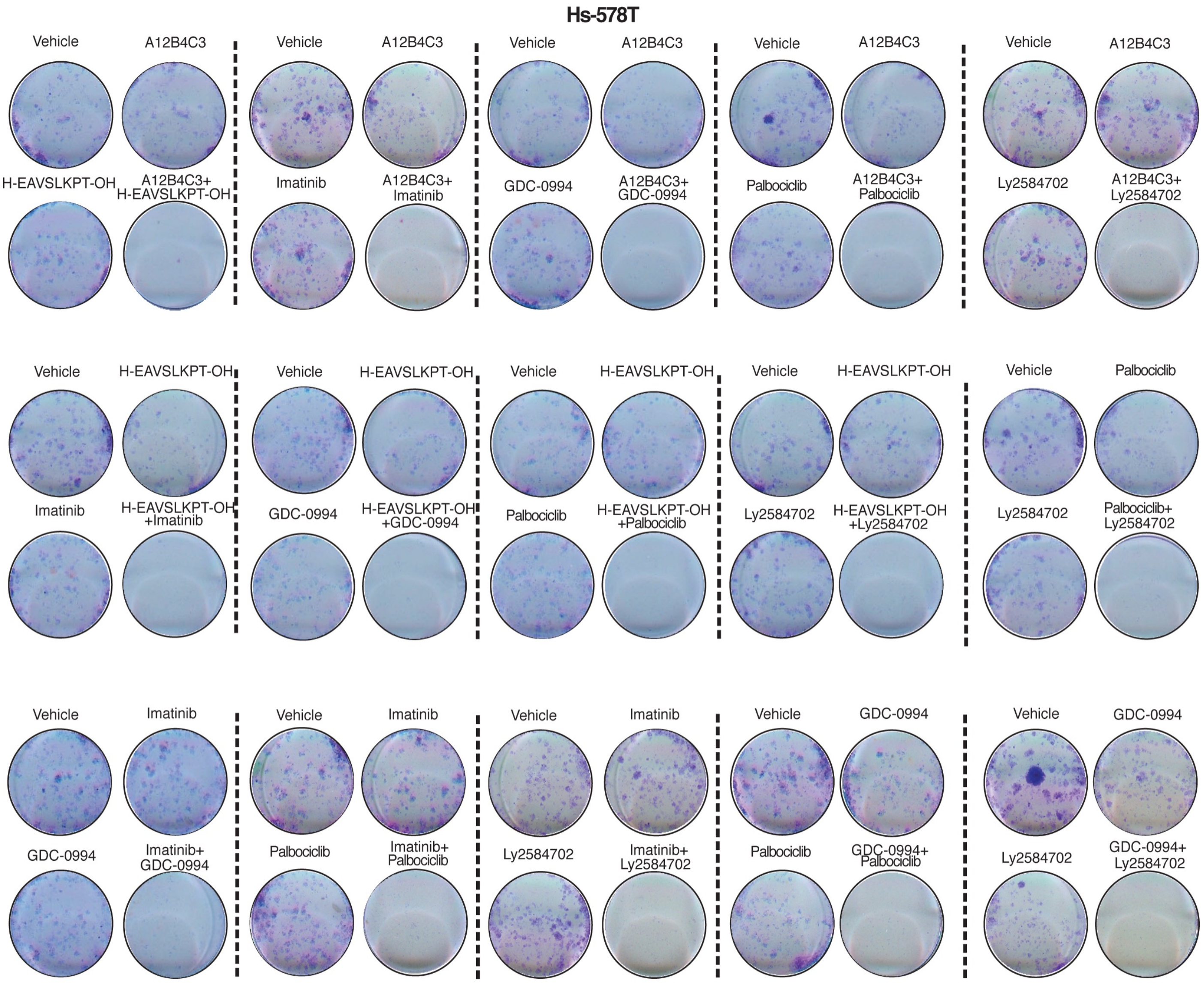

GDC-0994	62.5 nM
Imatinib	62.5 nM
A12B4C3	62.5 nM
Ly2584702	62.5 nM
H-EAVSLKPT-OH	62.5 nM
Palbociclib	8 nM

Supplementarty Figure 11. Colony assays (Hs-578T cell line). Colony assays showing the efficacy of vehicle or each of the six compounds targeting the six hyperactive kinases in K-high, alone or in combination (two drugs) against the aggressive TNBC cell line Hs-578T. Drugs were used at the depicted concentrations in the table. Representative images of three independent experiments.

MDA-MB-468

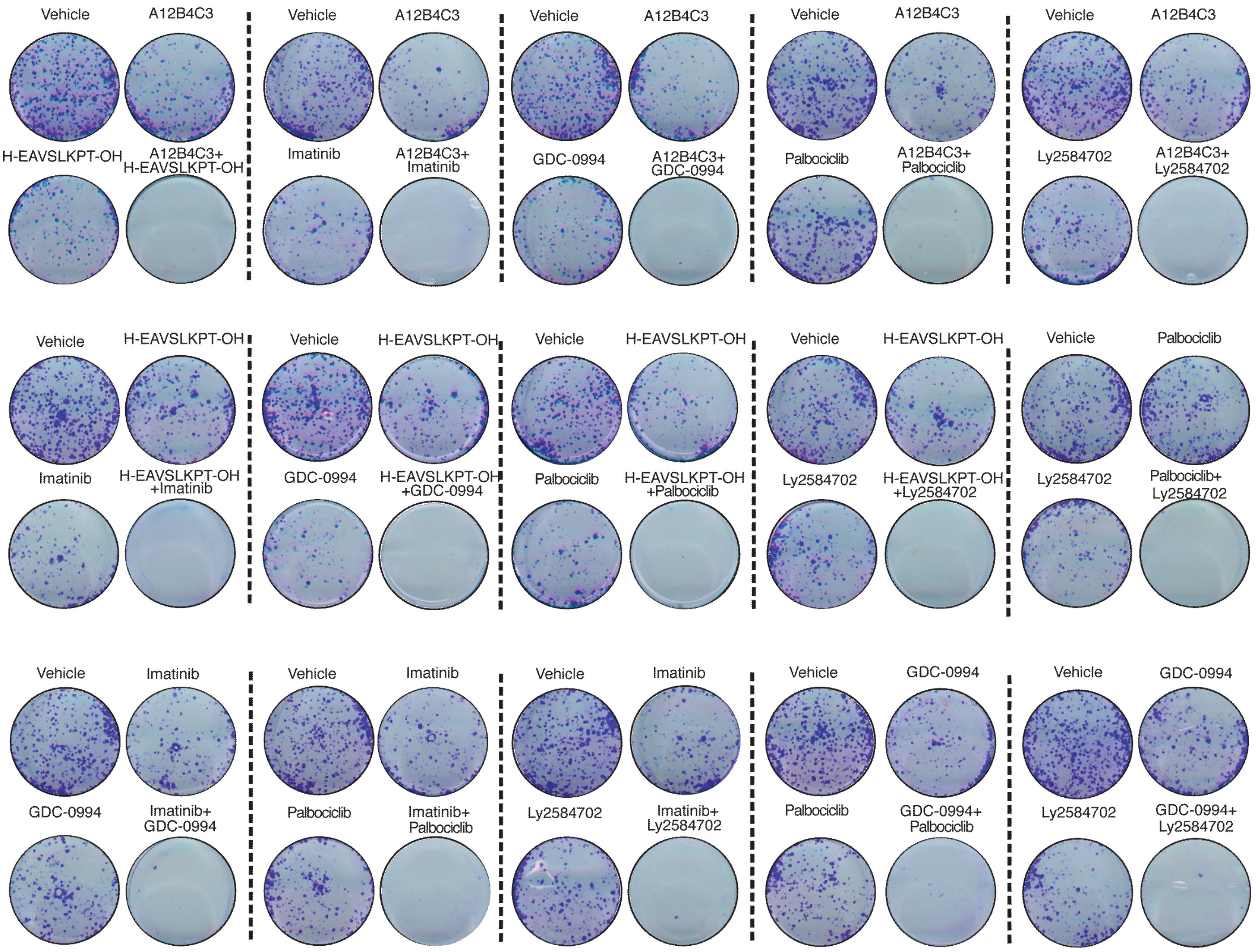

GDC-0994	31 nM
Imatinib	31 nM
A12B4C3	31 nM
Ly2584702	31 nM
H-EAVSLKPT-OH	31 nM
Palbociclib	4 nM

Supplementary Figure 12. Colony assays (MDA-MB-468 cell line). Colony assays showing the efficacy of vehicle or each of the six compounds targeting the six hyperactive kinases in K-high, alone or in combination (two drugs) against the TNBC cell line MDA-MB-468. Drugs were used at the depicted concentrations in the table. Representative images of three independent experiments.

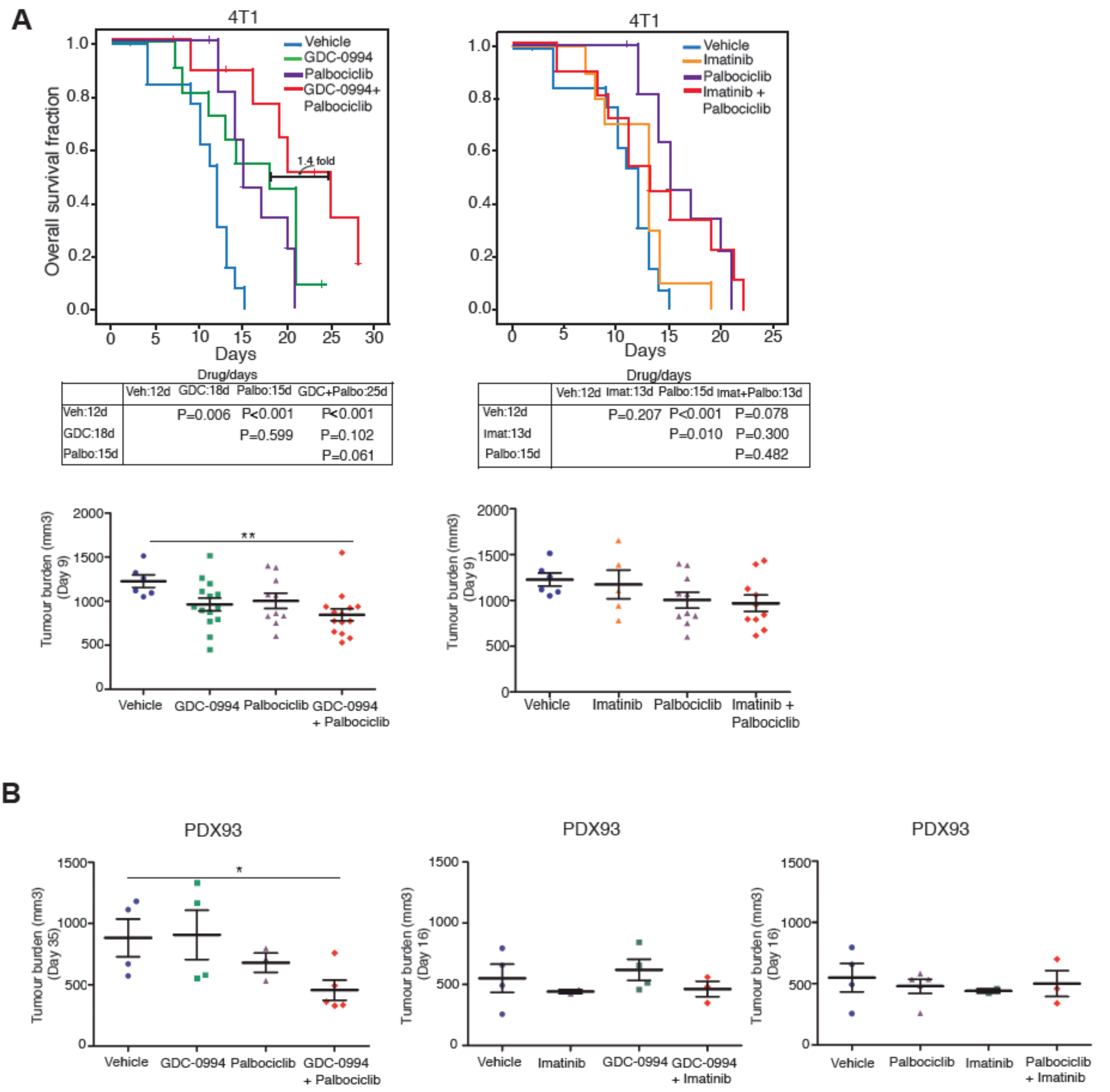

Supplementary Figure 13. *In vivo* efficacy in the 4T1 model and the human PDX93. (A) The 4T1 tumor model is a highly aggressive model of TNBC. Since it is a murine model, it preserves the immune system and shows natural growth kinetics; however, the evolution is very fast and it is considered a highly resistant tumour model. According to Figure 5, the targets that show the highest level of expression are p-ERK and CDK6. Congruently with this, only statistically significant differences in mice overall survival are observed when palbociclib and GDC-0994 are combined (left Kaplan-Meier chart), compared to each agent alone. Mice treated with: vehicle (n=14), imatinib (n=10), GDC-0994 (n=11), palbociclib (n=12), GDC-0994+palbociclib (n=14) and palbociclib+imatinib (n=11). **(B)** Finally, PDXs are thought to better recapitulate human tumours inter-patient heterogeneity. PDX93, a xenograft with high levels of p-ERK and CDK6 and low c-KIT (as opposed to PDX156; Figure 5), responds with tumor shrinkage to the combination of palbociclib and GDC-0994, but not to the combinations that include imatinib (as opposed to PDX156; Figure 6). Mice treated with: vehicle (n=3), imatinib (n=3), GDC-0994 (n=2), palbociclib (n=3), GDC-0994+palbociclib (n=3), GDC-0994+imatinib (n=2) and palbociclib+imatinib (n=3). In tumour burden graph, each point represents a tumour. The data are represented as mean ± SEM and Student ´s *t* test was performed. * $p<0.05$, ** $p<0.01$.

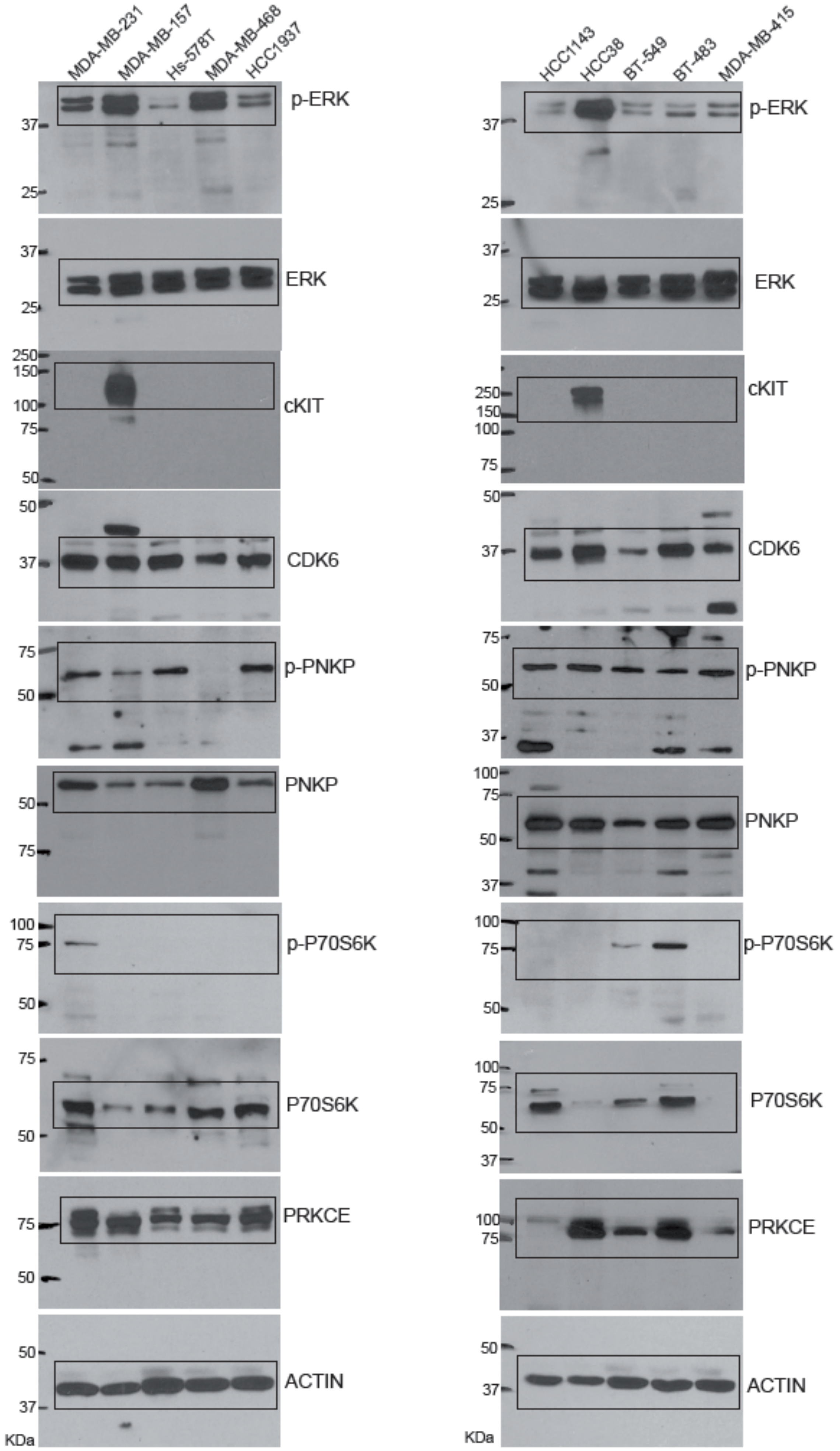

Supplementary Figure 14. Uncropped western blots from Figure 5, Panel A

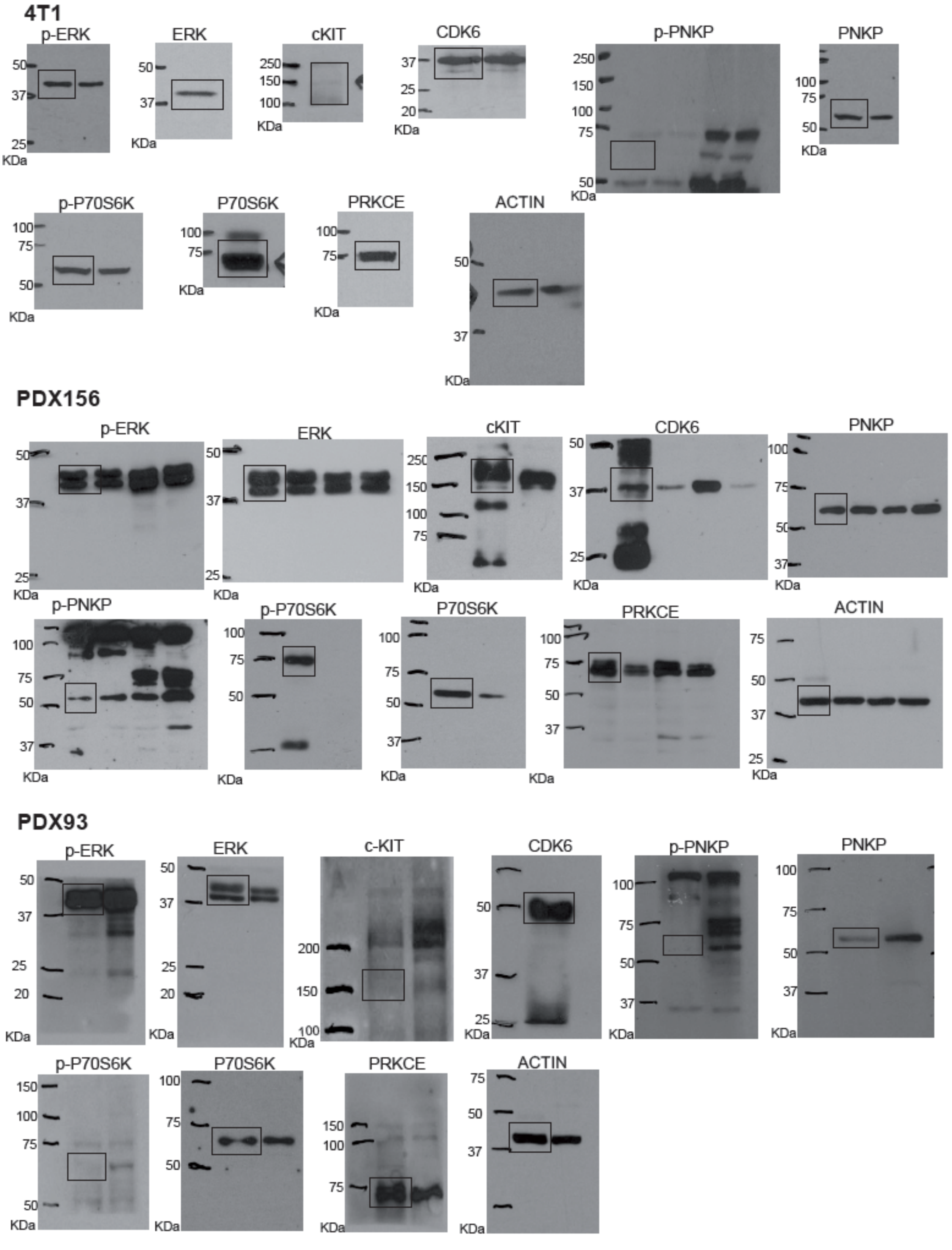

Supplementary Figure 15. Uncropped western blots from Figure 5, Panel A

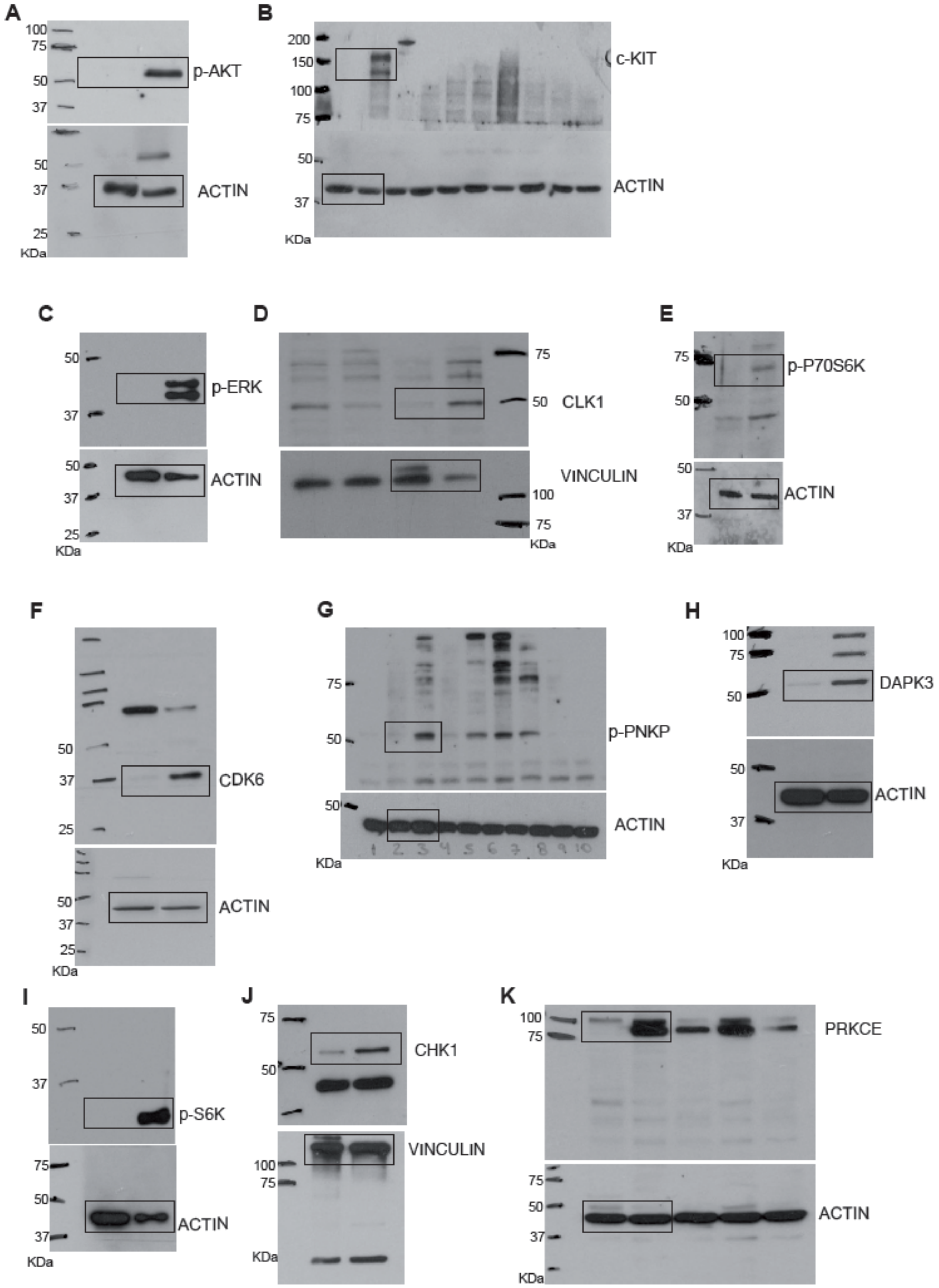

Supplementary Figure 16. Uncropped western blots from Supplementary Figure 3

Supplementary Tables

Supplementary Table 1. Clinical and demographic characteristics of the training and validation sets

	Training set (n = 34)		Validation sets (n = 174)	
	**Relapsed < 3 years (n = 13)**	**Nonrelapsed > 12 years (n = 21)**	**Val-1 (n = 113)**	**Val 2 (n = 61)**
**Age (years)** **Mean (range)**	54.1 (33-71)	58.2 (41-77)	56.7 (26-78)	54.1 (29-84)
**TNM stage**				
I	2 (15%)	4 (19%)	26 (23%)	14 (22.9%)
II	9 (69%)	13 (62%)	63 (55%)	35(57.4%)
III	2 (15%)	3 (19%)	24 (22%)	12 (19.7%)
**Type of Chemotherapy**				
None	0 (0%)	0 (0%)	12 (10%)	0 (0%)
CMF	0 (0%)	0 (0%)	33 (29%)	0 (0%)
Cyclophosph. + Anthrac.	3 (23%)	4 (19%)	41 (36%)	0 (0%)
Cyclophosph. + anthrac. + taxane	10 (77%)	17 (81%)	27 (25%)	61 (100%)
**Ki67 average (range)**	39 (3-92%)	42 (2-89%)	39.8% (1-97%)	40% (2-98%)
**Grade**				
G1	0 (0%)	0 (0%)	4 (3%)	0 (0%)
G2	5 (38%)	4 (19%)	31 (27%)	18 (29.5%)
G3	8 (61%)	17 (81%)	78 (70%)	43 (70.5%)
**Premenopausal**	9 (70%)	17 (81%)	43 (38%)	20 (32.8%)

Supplementary Table 2. PSM descriptions of mass spectrometry runs assaying tumour samples and cell lines

	Tumour samples	Cell lines	Combined*
**MS/MS**	1487610	814774	2302384*
**PSMs**	254204	200489	454074*
**Phospho PSMs**	237212	180185	417076*
**% Enrichment**	93.3%	90%	92%
**Unique phospho-peptides**	9008	10380	
**Phosphoproteins**	2643	3100	3634
**Phosphosites**	10080	11964	15160
**Serine+**	8538	10047	12638
**Threonine+**	1381	1768	2301
**Tyrosine+**	161	149	221
**Mono-phosphorylated**	4717	6530	7212
**Bi-phosphorylated**	5650	5060	7462
**Tri-/tetra-/penta-phosphorylated**	1204	803	1545

*The numbers in the first three rows are the results of the addition of the raw spectra captured or identified globally in the experiment, but broken down depending on whether they were derived from runs of the cell lines or tumour samples. However, they correspond to pre-identification data. After identification (unique phosphopeptides, unique proteins), the data from the "combined" column correspond to total nonredundant elements (10080 and 11964 phosphosites were identified, respectively, in tumour samples and cell lines; however, the total number of unique phosphosites in the experiment was 15160, since many of them were common to both the cell lines and the tumour samples).

+Compared with the reported ratio of phosphosites in the human cell proteome (1800Ser:200Thr:1Tyr[14, 15]), we observed a slight bias towards an increase in phosphotyrosines (12638 were serine, 2301 threonine, and

221 tyrosine residues). However, the reported ratios are average ratios, and specific changes such as signalling aberrations in cancer tissues or variations between different cell lineages may be observed.

Supplementary Table 3. H-score quartiles for each tested probe

Probe	Upper quartile (75)	Medium quartile (50)	Lower quartile (25)
**CDK6**	0.0783	0.0280	0.0073
**pPNKP Ser114/Thr118**	0.6968	0.4562	0.2464
**c-Kit**	0.0057	0.0029	0.0011
**PRKCE**	0.1754	0.0515	0.0170
**pERK Thr202/Tyr204**	0.4075	0.1099	0.0168
**pP70S6K Thr389**	0.2728	0.1383	0.0295
**CHK1**	0.8167	0.5581	0.2126
**pAKT Ser473**	0.2206	0.1084	0.0365
**CLK1**	1.0049	0.7888	0.6007
**pS6K Ser240/Ser244**	0.2395	0.1048	0.0406
**DAPK3**	0.9532	0.7504	0.5642

Supplementary References

1. Alessi DR, *et al.* Mechanism of activation of protein kinase B by insulin and IGF-1. *EMBO J* **15**, 6541-6551 (1996).

2. Warfel NA, Kraft AS. PIM kinase (and Akt) biology and signaling in tumors. *Pharmacol Ther* **151**, 41-49 (2015).

3. Hirota S*, et al.* Gain-of-function mutations of c-kit in human gastrointestinal stromal tumors. *Science* **279**, 577-580 (1998).

4. Gorin MA, Pan Q. Protein kinase C epsilon: an oncogene and emerging tumor biomarker. *Mol Cancer* **8**, 9 (2009).

5. Johnson JE, Giorgione J, Newton AC. The C1 and C2 domains of protein kinase C are independent membrane targeting modules, with specificity for phosphatidylserine conferred by the C1 domain. *Biochemistry* **39**, 11360-11369 (2000).

6. Cenni V, Doppler H, Sonnenburg ED, Maraldi N, Newton AC, Toker A. Regulation of novel protein kinase C epsilon by phosphorylation. *Biochem J* **363**, 537-545 (2002).

7. Nakahara M*, et al.* A novel gain-of-function mutation of c-kit gene in gastrointestinal stromal tumors. *Gastroenterology* **115**, 1090-1095 (1998).

8. Lasota J, Jasinski M, Sarlomo-Rikala M, Miettinen M. Mutations in exon 11 of c-Kit occur preferentially in malignant versus benign gastrointestinal stromal tumors and do not occur in leiomyomas or leiomyosarcomas. *Am J Pathol* **154**, 53-60 (1999).

9. Sattler M*, et al.* Steel factor induces tyrosine phosphorylation of CRKL and binding of CRKL to a complex containing c-kit, phosphatidylinositol 3-kinase, and p120(CBL). *J Biol Chem* **272**, 10248-10253 (1997).

10. Blume-Jensen P, Jiang G, Hyman R, Lee KF, O'Gorman S, Hunter T. Kit/stem cell factor receptor-induced activation of phosphatidylinositol 3'-kinase is essential for male fertility. *Nat Genet* **24**, 157-162 (2000).

11. Miller ML*, et al.* Linear motif atlas for phosphorylation-dependent signaling. *Sci Signal* **1**, ra2 (2008).

12. Linding R*, et al.* Systematic discovery of in vivo phosphorylation networks. *Cell* **129**, 1415-1426 (2007).

13. She QB*, et al.* 4E-BP1 is a key effector of the oncogenic activation of the AKT and ERK signaling pathways that integrates their function in tumors. *Cancer Cell* **18**, 39-51 (2010).

14. Hunter T. Tyrosine phosphorylation in cell signaling and disease. *Keio J Med* **51**, 61-71 (2002).

15. Ye J*, et al.* Optimized IMAC-IMAC protocol for phosphopeptide recovery from complex biological samples. *J Proteome Res* **9**, 3561-3573 (2010).
