# Supplementary material for: In vivo phosphoproteomics reveals kinase activity profiles that predict treatment outcome in triple-negative breast cancer
Source: Nat Commun. 2018 Aug 29;9:3501. doi: 10.1038/s41467-018-05742-z (PMC6115463; doi:10.1038/s41467-018-05742-z)
Supplement: Supplementary file 3 — Supplementary Data 1 [file 41467_2018_5742_MOESM3_ESM.pdf]

Description of Additional Supplementary Files

File Name: Supplementary Data 1
Description: For each of the 159 peptides upregulated in the relapsed cases in the training set, the following data are provided: their mapping protein, the phosphorylated residue(s), the kinases whose activity determines phophorylation status, and the absolute intensity and presence/absence in the runs of relapsed and non-relapsed cases.

File Name: Supplementary Data 2
Description: Kinase activation patterns found in Val-1: description, number of patient tumours with the pattern, and number of patient tumours with the patterns that relapsed or were cured.

File Name: Supplementary Data 3
Description: Kinase activation patterns found in Val-2: description, number of patient tumours with the pattern, and number of patient tumours with the patterns that relapsed or were cured. The last column details whether each kinase activation pattern was also found in Val-1 (the majority of the patterns) or whether it was discovered only in Val-2.

File Name: Supplementary Data 4
Description: Indexing primers used to amplify captured DNA libraries.
