# Supplementary material for: In vivo phosphoproteomics reveals kinase activity profiles that predict treatment outcome in triple-negative breast cancer
Source: Nat Commun. 2018 Aug 29;9:3501. doi: 10.1038/s41467-018-05742-z (PMC6115463; doi:10.1038/s41467-018-05742-z)
Supplement: Supplementary file 2 — Description of Additional Supplementary Files [file 41467_2018_5742_MOESM2_ESM.pdf]

Editorial Note 1: This manuscript has been previously reviewed at another journal that is not operating a transparent peer review scheme. This document only contains reviewer comments and rebuttal letters for versions considered at Nature Communications.

Editorial Note 2: Parts of this peer review file have been redacted as indicated to maintain confidential information

Answer to Reviewers' Comments

Reviewer #1:

Remarks to the Author:

[redacted]

The authors have addressed most of reviewer's questions and thoroughly amended the manuscript, in particular, they have:

- Corroborated the follow up therapeutic data, including PDX assays;
- Repeated all in vitro cytotoxicity assays, with more appropriate drug concentrations ad readout (e.g. the colony assays);
- Addressed various technical issues, concerning both wet-experiments (e.g. the HICs) and data analysis, with additional and clearer displays that facilitate the comprehension of the data;
- Added more detailed explanation concerning the choice of the patient cohorts analysed;
- Incudes an additional validation set (Val-2) of early TNBC patients, which was very useful to tackle some of the concerns raised by reviewers (e.g. the type of tenement received by the patients, not anymore reflecting the state-of-the-art)

In addition, they are toned-down the numerous overstatements of the first version of the manuscript, highlighting now that the study value resides mainly in being a proof-of-concept of the power of molecular phosphorylation profiling in cancer patient samples, to define novel molecular signatures which may help prediction of treatment outcome. Last, figures are now much clearer and convincing.

Taking into consideration all the efforts put in the revision, the much-improved outline and the more robust data, the different audients, scope and audience the new journal to which the manuscript is submitted, we are pleased to confirm that in its present from, this paper is eligible for publication in Nature Communications, where we are confident it will raise considerable interest.

A few minor points:

- c-Kit is not expressed in the cell lines that were tested in Figure 5 and Figures S11/12, but the compound that is used to inhibit this kinase, Imatinib, has nevertheless an effect in all the combination tested. The authors should comment on this result, and consider possible effects mediated by other kinases that are targeted by the compound.

We have added the following sentence right before the study limitations in the discussion section.

Nevertheless, the detected efficacy of imatinib in models with little c-Kit levels suggest either certain off-target effect of the agent or, most likely, that the specificity is not 100%.

- PNKP deletion provides an advantage in MDA-MB-231 cells but not in MDA-MB-468 cells, which show low p-PNKP. Because this is a nice piece of information, some of the results shown in figure S13 could be included in main Figure 6.

Data have been re-arranged according to this suggestion.

- page 9, lanes 242-242: In the sentence "However, in PDX93, only palbociclib plus imatinib, but not the imatinib-based doublets, achieved statistically significant differences in tumor control (Figure S14B)", it seems like "imatinib" should be replaced with "GDC-0994". In addition, in figure S14B no significance is reported for any of the combinations.

We have corrected "imatinib" for "GDC-0994", that is the correct drug name. We thank the reviewer for noticing this mistake.

Reviewer #2:

Remarks to the Author:

The manuscript has improved tremendously over the initial version and I now support publication.
